# Comparison of Polypentenamer and Polynorbornene Bottlebrushes in Dilute Solution

**DOI:** 10.1021/acspolymersau.3c00052

**Published:** 2024-02-24

**Authors:** Courtney M. Leo, Jaehoon Jang, Ethan J. Corey, William J. Neary, Jared I. Bowman, Justin G. Kennemur

**Affiliations:** † Department of Chemistry and Biochemistry, Florida State University, Tallahassee, Florida 32303, United States; ‡ Department of Chemistry, University of California at Riverside, Riverside, California 92521, United States; § George and Josephine Butler Polymer Research Laboratory, Center for Macromolecular Science & Engineering, Department of Chemistry, University of Florida, Gainesville, Florida 32611, United States

**Keywords:** bottlebrush, dilute solution, ROMP, ATRP, grafting-from, viscosity

## Abstract

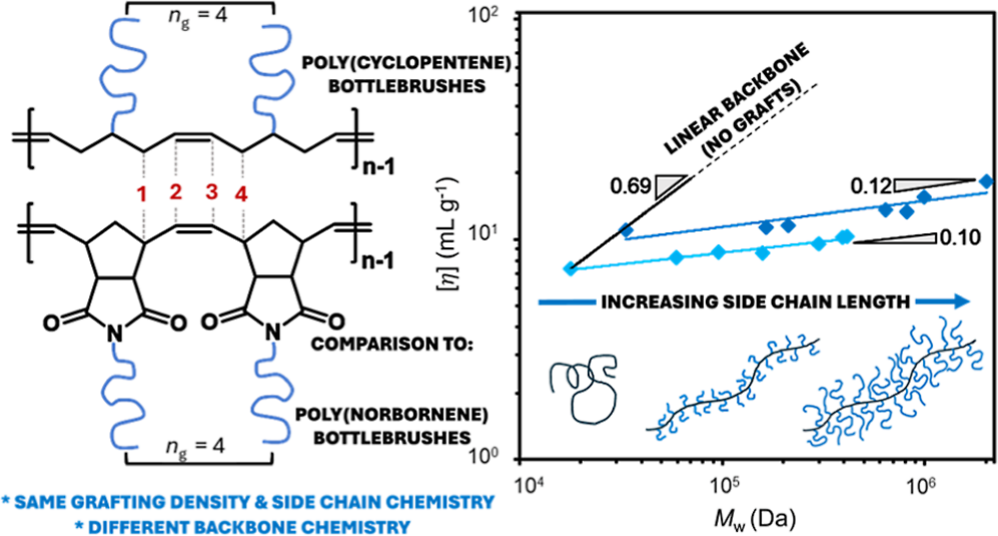

Bottlebrush (BB) polymers were synthesized via grafting-from-atom transfer radical polymerization (ATRP) of styrene on polypentenamer and polynorbornene macroinitiators with matched grafting density (*n*_g_ = 4) and backbone degrees of polymerization (122 ≥ *N*_bb_ ≥ 61) to produce a comparative study on their respective dilute solution properties as a function of increasing side chain degree of polymerization (116 ≥ *N*_sc_ ≥ 5). The grafting-from technique produced near quantitative grafting efficiency and narrow dispersity *N*_sc_ as evidenced by spectroscopic analysis and ring closing metathesis depolymerization of the polypentenamer BBs. The versatility of this synthetic approach permitted a comprehensive survey of power law expressions that arise from monitoring intrinsic viscosity, hydrodynamic radius, and radius of gyration as a function of increasing the molar mass of the BBs by increasing *N*_sc_. These values were compared to a series of linear (nongrafted, *N*_sc_ = 0) macroinitiators in addition to linear grafts. This unique study allowed elucidation of the onset of bottlebrush behavior for two different types of bottlebrush backbones with identical grafting density but inherently different flexibility. In addition, grafting-from ATRP of methyl acrylate on a polypentenamer macroinitiator allowed the observation of the effects of graft chemistry in comparison to polystyrene. Differences in the observed scaling relationships in dilute solution as a function of each of these synthetic variants are discussed.

## Introduction

Well-defined and densely grafted brush polymers have received increased attention over the last few decades because they display properties significantly different from those of traditional branched or linear polymers. The dense grafting of side chains distinguishes this subclass of macromolecules, known as bottlebrushes (BBs), from other branched or comb polymers due to an unorthodox combination of high molar mass yet low viscosity.^1−3^ This behavior is attributed to steric repulsions between neighboring side chains, which occupy the excluded volume near the backbone and increase the BB persistence length. The BB molecular architecture is inspired by naturally occurring proteoglycans present in biological constructs, such as articular cartilage, where lubrication and impact damping are required.^4,5^ BB syntheses have progressed rapidly since their introduction,^1,3,6−20^ showing promise in applications such as super soft gels/elastomers,^21−23^ drug delivery agents^24−26^ photonic crystals,^27,28^ energy storage,^29^ and advanced nanoscale patterning.^30−32^ Despite recent experimental and theoretical efforts, distinct relationships between BB polymer architecture and material properties are still ongoing due to the many modifiable architectural parameters. These include the chemical composition of the backbone and side chains, the number of backbone atoms between side chains (*n*_g_), the side chain degree of polymerization (*N*_sc_), and the backbone degree of polymerization (*N*_bb_).

Several experimental, theoretical, and simulation studies have focused on BBs in the melt state,^33−47^ while other studies have focused on their dilute solution properties.^48−62^ Typical parameters used to characterize molecular structure in dilute solution include intrinsic viscosity [η], hydrodynamic radius (*R*_h_), and radius of gyration (*R*_g_). Combined, these parameters provide sensitized information on the size and shape of a polymer in a chosen solvent/temperature as architectural features are systematically changed.

**Figure 1:**
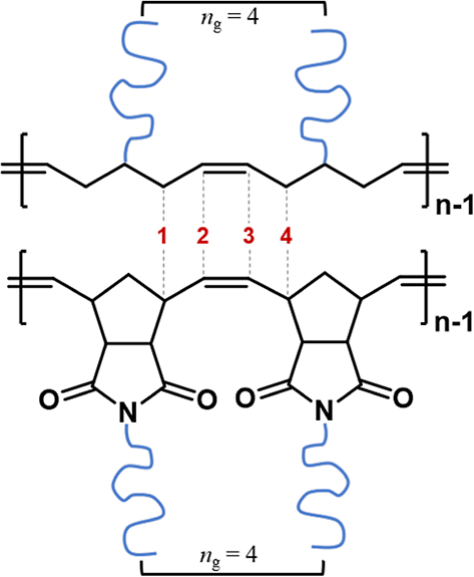
Structural comparison of bottlebrushes synthesized with a PCP (top) or maleimide-fused PNB (bottom) backbone. Both microstructures have identical graft densities (*n*_g_ = 4).

To date, polynorbornene (PNB)-based BBs have received the most attention in experimental and theoretical studies due to their modular assembly.^63^ A norbornene (NB) macromonomer (MM) can be prepared with a variety of side chain chemistries and subsequently grafted-through by ring-opening metathesis polymerization (ROMP).^64^ The high ring strain energy (∼67 kJ mol^–1^)^65^ of NB can be exploited to yield high conversion and quantitative grafting efficiency, making this approach a choice method of BB synthesis. Since PNB-based BBs typically present one graft per five-carbon repeating unit (*n*_g_ = 4), this grafting density is generally accepted as suitable to produce BB behavior. However, the PNB repeating unit uniquely features carbons that are conformationally hindered either by sp^2^ hybridization (olefin) or by part of a cyclopentane ring (Figure 1). Therefore, questions still remain surrounding the inherent stiffness of the PNB backbone and its role within BB properties. For example, PNB has a glass transition temperature (*T*_g_) of ∼45 °C^66^ (depending on stereochemistry) which is ∼90 °C higher than polycyclopentene (PCP),^67^ an analogous five-carbon repeating unit without a cyclopentane ring tethered within the backbone. In many BB syntheses, the PNB backbone is often further sterically encumbered through the use of a fused maleimide heterocycle with an *N*-pendant attachment of the side chain (Figure 1). The consequence of this additional encumbrance is illustrated by the increase in *T*_g_ (∼233 °C) of poly(*exo*-*N*-phenylnorbornene-5,6-dicarboximide),^68^ while a phenyl pendant on the repeating unit of PCP results in a *T*_g_ ∼ 17 °C.^69,70^

**Scheme 1:**
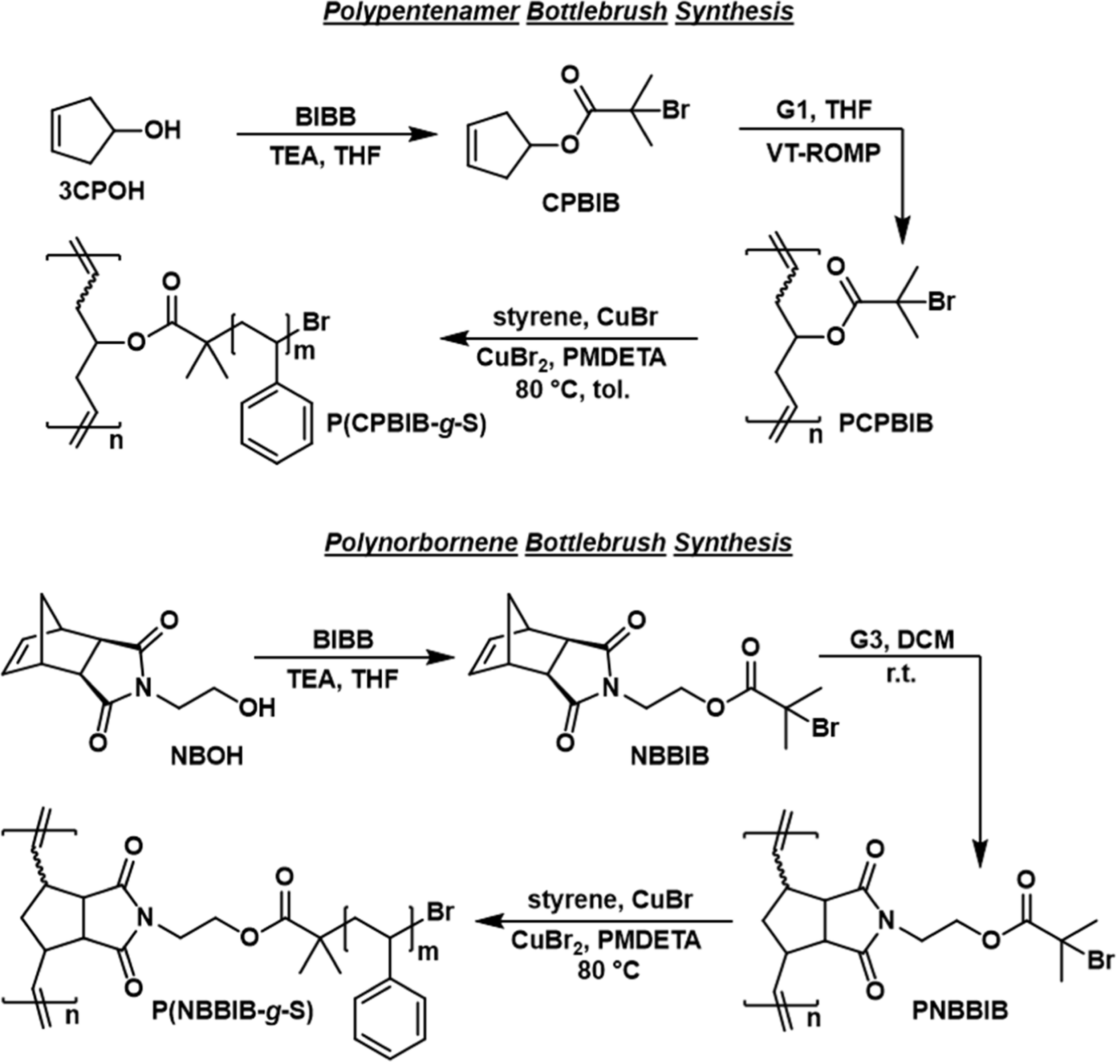
Synthesis of PCP and PNB Bottlebrushes

For several years, our group has investigated the design and unique properties afforded by PCP derivatives. To expand the limited suite of BB backbone chemistries available for probing structure–property relationships, we recently reported the synthesis of well-defined PCP-based BBs via variable–temperature ROMP (VT-ROMP) of α-bromoisobutyryl bromide (BIBB) functionalized cyclopent-3-en-1-ol (CP3OH) and subsequent grafting-from by atom transfer radical polymerization (ATRP) of styrene (S) (Scheme 1).^71^ A high degree of control over *N*_bb_, *N*_sc_, and dispersity (*D̵*), as well as quantitative grafting efficiency was achieved. Furthermore, side chain *N*_sc_ and *D̵* following grafting-from were able to be verified by quantitative ring-closing metathesis depolymerization (RCMD) of the PCP backbone.^72^ These alternative BB systems have the potential to improve our understanding of structure–property relationships associated with these complex architectures when the PNB backbone is uniquely replaced with a more flexible PCP alternative.

In this work, we compare the dilute solution properties of several sets of PNB and PCP BB polymers as a function of both *N*_bb_ and *N*_sc_. Values and scaling relationships for intrinsic viscosity [η], radius of hydration (*R*_h_), and radius of gyration (*R*_g_) are gathered in this study that uniquely compares two different BB backbone chemistries with matched *n*_g_ to observe differences in BB behavior that can be directly correlated to the backbone as a function of *N*_sc_. We also compare two different side chain chemistries from S and methyl acrylate (MA) on the PCP backbone. The measured BB properties were also compared to linear analogs of the backbones and side chains for control analysis.

## Experimental Section

### Bottlebrush Synthesis

The PCP and PNB macroinitiators, each functionalized with one BIB pendant per five-carbon repeating unit (*n*_g_ = 4), were synthesized via ROMP using Grubbs first-generation (G1) or third-generation (G3) catalyst (pyridine adduct), respectively, according to previous literature (Scheme 1). For each macroinitiator, standard ATRP methods were used for grafting from a targeted *N*_sc_ using either S or MA with CuBr/CuBr_2_, *N*,*N*,*N*′,*N*″,*N*″-pentamethyl diethylenetriamine (PMDETA) ligand, and toluene or anisole solvent. Detailed synthetic procedures and characterizations can be found in the Supporting Information document. For each macroinitiator and BB sample ID (Tables 1–4), the numbers in parentheses indicate the number-average degree of polymerization (*N*_n_). For example, PCPBIB(111)-*g*-S(10) has *N*_bb_ = 111 and *N*_sc_ = 10.

**Table 1:** Characterization Data for Linear PCP and PNB Macroinitiators and PS Side Chains

sample ID^a^	*M*_n,MALS_^b^ (kDa)	*M*_w,MALS_^b^ (kDa)	*Đ*^b^	[η] (mL g^– 1^)	*R*_h_ (nm)
PCPBIB(61)	14.2	17.9	1.23	10.3	3.0
PCPBIB(111)	26.0	33.5	1.29	15.5	4.3
PCPBIB(133)	31.3	39.1	1.25	18.3	4.7
PCPBIB(173)	40.3	51.1	1.27	21.6	5.4
PCPBIB(225)	52.5	69.8	1.33	26.0	6.5
PNBBIB(68)	24.2	24.4	1.01	8.9	3.2
PNBBIB(83)	29.7	30.0	1.01	11.0	3.7
PNBBIB(109)	38.9	39.3	1.01	12.6	4.3
PNBBIB(122)	43.5	43.5	1.00	13.2	4.5
PNBBIB(183)	65.1	65.8	1.01	17.9	5.7
PNBBIB(239)	85.0	88.4	1.04	20.0	6.4
CP-PS(28)^c^	3.1	3.3	1.05	4.3	1.3
CP-PS(55)^c^	6.0	6.1	1.02	6.3	1.8
CP-PS(73)^c^	7.8	7.9	1.01	6.7	2.0
CP-PS(108)	11.5	12.0	1.04	9.2	2.6
CP-PS(191)	20.3	20.3	1.00	13.3	3.5
CP-PS(280)	29.4	29.9	1.02	17.5	4.4
CP-PS(406)	42.5	42.9	1.01	21.6	5.3
CP-PS(578)	60.4	64.3	1.06	28.7	6.6

a Number in parentheses is the number-average degree of polymerization.

b Determined by MALS-SEC (THF, 25 °C) using a d*n*/d*c* value of 0.090, 0.107, and 0.185 mL g^–1^ for PCPBIB, PNBBIB, and CP-PS, respectively.

c Produced from RCMD of the PCPBIB(111)-*g*-S series.

**Table 2:** Characterization Data for PCPBIB-*g*-S Bottlebrushes

sample ID^a^	*M*_n,MALS_^b^ (kDa)	*M*_w,MALS_^b^ (kDa)	*Đ*^b^	[η] (mL g^– 1^)	*R*_g_ (nm)	*R*_h_ (nm)	*p*^c^
PCPBIB(61)-*g*-S(5)	48.8	59.3	1.22	11.5	10.6	4.7	2.3
PCPBIB(61)-*g*-S(10)	76.5	96.1	1.26	12.3	12.2	5.6	2.2
PCPBIB(61)-*g*-S(18)	125.9	157.8	1.25	12.1	16.1	6.6	2.4
PCPBIB(61)-*g*-S(32)	220	284.0	1.29	13.3	15.5	8.4	1.9
PCPBIB(61)-*g*-S(45)	298.8	393.1	1.32	14.2	17.0	9.4	1.8
PCPBIB(61)-*g*-S(49)	324.9	415.1	1.28	14.5	16.3	9.6	1.7
PCPBIB(111)-*g*-S(8)	122.7	164.5	1.34	15.9	12.3	7.2	1.7
PCPBIB(111)-*g*-S(11)	156.5	212.0	1.35	16.3	10.8	8.0	1.4
PCPBIB(111)-*g*-S(33)	412.3	637.2	1.55	19.1	18.9	11.9	1.6
PCPBIB(111)-*g*-S(49)	597.3	812.9	1.36	18.7	17.8	13.8	1.3
PCPBIB(111)-*g*-S(60)	724.8	989.4	1.37	21.8	21.0	14.6	1.4
PCPBIB(111)-*g*-S(116)	1372.6	2029.3	1.48	25.6	32.2	19.4	1.7

a Numbers in parentheses are the number-average degree of polymerizations.

b Determined by MALS-SEC (THF, 25 °C) using a d*n*/d*c* value corrected by the weight fraction of PS (0.185 mL g^–1^) and PCPBIB (0.090 mL g^–1^).

c Shape factor calculated as (*R*_g_/*R*_h_).

**Table 3:** Characterization Data for PNBBIB-*g*-S Bottlebrushes

sample ID^a^	*M*_n,MALS_^b^ (kDa)	*M*_w,MALS_^b^ (kDa)	*Đ*^b^	[η] (mL g^– 1^)	*R*_g_ (nm)	*R*_h_ (nm)	*p*^c^
PNBBIB(68)-g-S(5)	62.4	62.7	1.01	10.2	5.2	4.7	1.1
PNBBIB(68)-g-S(18)	152.6	155.0	1.02	11.8	6.6	6.6	1.0
PNBBIB(68)-g-S(23)	186.4	190.3	1.02	12.3	7.2	7.2	1.0
PNBBIB(68)-*g*-S(26)	205.3	206.7	1.01	12.6	7.9	7.4	1.1
PNBBIB(68)-*g*-S(39)	297.2	299.9	1.01	12.9	8.5	8.5	1.0
PNBBIB(68)-*g*-S(43)	325.4	329.2	1.01	14.1	10.0	9.0	1.1
PNBBIB(68)-*g*-S(56)	422.4	425.1	1.01	14.9	9.0	10.0	0.9
PNBBIB(122)-*g*-S(7)	130.7	135.7	1.04	18.7	12.9	7.4	1.7
PNBBIB(122)-*g*-S(11)	182.2	192.1	1.05	19.0	7.9	8.2	1.0
PNBBIB(122)-*g*-S(21)	305.4	312.6	1.02	20.8	16.3	10.1	1.6
PNBBIB(122)-*g*-S(45)	616.6	650.2	1.05	23.2	15.6	13.3	1.2
PNBBIB(122)-*g*-S(53)	712.1	764.4	1.07	24.3	19.0	14.2	1.3
PNBBIB(122)-*g*-S(61)	817.2	893.5	1.09	24.8	19.7	15.1	1.3

a Numbers in parentheses are the number-average degree of polymerizations.

b Determined by MALS-SEC (THF, 25 °C) using a d*n*/d*c* value corrected by the weight fraction of PS (0.185 mL g^–1^) and PNPBIB (0.107 mL g^–1^).

c Shape factor calculated as (*R*_g_/*R*_h_).

**Table 4:**
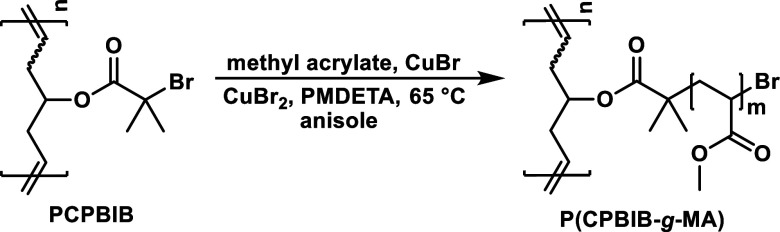
(Top) Synthesis and (Bottom) Tabulated Characterization Data for PCPBIB(61)-*g*-MA Bottlebrushes

sample ID^a^	*M*_n,MALS_^b^ (kDa)	*M*_w,MALS_^b^ (kDa)	*Đ*^b^	[η] (mL g^– 1^)	*R*_g_ (nm)	*R*_h_ (nm)	*p*^c^
PCPBIB(61)-*g*-MA(11)	72.3	109.8	1.52	14.8	13.0	6.1	2.1
PCPBIB(61)-*g*-MA(17)	105.5	155.2	1.47	16.6	11.6	7.1	1.6
PCPBIB(61)-*g*-MA(21)	123.7	183.6	1.48	16.9	13.0	7.6	1.7
PCPBIB(61)-*g*-MA(26)	152.8	211.0	1.38	17.9	14.9	8.2	1.8
PCPBIB(61)-*g*-MA(48)	266.4	381.1	1.43	21.4	16.7	10.5	1.6
PCPBIB(61)-*g*-MA(57)	334.9	535.9	1.60	23.9	18.8	12.0	1.6
PCPBIB(61)-*g*-MA(72)	393.8	626.4	1.59	25.4	22.7	12.9	1.8

a Numbers in parentheses are the number average degrees of polymerizations.

b Determined by MALS-SEC (THF, 25 °C) using a d*n*/d*c* value corrected by the weight fraction of PMA (0.084 mL g^–1^) and PCPBIB (0.090 mL g^–1^).

c Shape factor calculated as (*R*_g_/*R*_h_).

### Size-Exclusion Chromatography

Two independent size exclusion chromatography (SEC) systems were used to analyze and cross-check the dilute solution properties of the BBs. Both instruments featured an Agilent–Wyatt combination SEC containing an Agilent 1260 Infinity model isocratic pump, degasser, autosampler, and thermostated column chamber. The Wyatt triple detection systems were composed of a miniDAWN TREOS 3-angle light scattering detector with 60 mW laser power, an Optilab TrEX refractive index detector, and a Viscostar II 4-capillary differential viscometer. On one SEC, the columns used were a Viscogel I-series 5 μm guard column followed by two sequential ViscoGel I-series G3078 mixed-bed columns. In the other SEC, two sequential Tosoh TSK Gel GMHhr-M, 5 μm mixed bed, 7.7 mm IDx30 cm were employed. The mobile phase used in both instruments was THF with a column temperature of 25 °C. A specific refractive index increment (d*n*/d*c*) of 0.0902 mL g^–1^ for PCPBIB and 0.107 mL g^–1^ for PNBBIB was obtained using a Wyatt injection system high-pressure (WISH) kit (1 mL loop) by analyzing the change in refractive index at various known concentrations of each macroinitiator in THF at 25 °C. The absolute molar mass (*M*_w_) of the BBs was calculated through the Wyatt ASTRA data acquisition software using sample solutions at a concentration of ∼5 mg mL^–1^ in the THF mobile phase. The d*n*/d*c* values of BBs samples were corrected using the weight fraction of PS (d*n*/d*c*_(25 °C, THF)_ = 0.185 mL g^–1^) or PMA (d*n*/d*c*_(25 °C, THF)_ = 0.084 mL g^–1^). The weight fractions were determined via ^1^H NMR as previously reported.^71^ All *M*_w_ measurements assumed 100% mass elution from the columns. The entirety of PCPBIB(61)-*g*-MA and PNBBIB(122)-*g*-S BB sets were analyzed on one SEC system and PCPBIB(61)-*g*-S, PCPBIB(111)-*g*-S, and PNBBIB(68)-*g*-S were analyzed via another, with select samples analyzed on both for control. While slight discrepancies in absolute values of solution property measurements were observed between the two SEC systems, the relative scaling of entire BB sets was consistent between the two instruments.

## Results and Discussion

Historically, dilute solution studies and the resulting properties have focused on comb or branched polymers with limited graft density.^48,59,61,73−78^ More recently, densely grafted systems, such as BBs, have received increased attention due to their unique properties. Many of these studies have focused on BBs with backbones produced from vinyl monomers (e.g., acrylates or styrenics) that harbor diverse side-chain chemistries at a near maximum grafting density limit of every other backbone carbon (*n*_g_ = 1).^58,60,79−81^ Singular or combined studies utilizing small angle neutron scattering (SANS), static light scattering (SLS), viscometry, and molecular dynamic (MD) simulations have provided the field with important scaling relationships, backbone persistence lengths, and molecular Kuhn lengths when diluted in solvents of varying quality. A pertinent and recent study by López-Barrón focused on a homologous series of poly(α-olefin)s with branch lengths up to 16 carbons long to explore dilute solution properties of BBs with *n*_g_ = 1 and within the lower limit of *N*_sc_ (i.e., an *n*-alkyl branch of 1–16 carbons).^60^ A notable observation was that the power law dependence of intrinsic viscosity (eq 1)
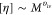
1where *M* is molar mass and υ_iv_ is the scaling exponent showed a decrease in υ_iv_ up to ∼*N*_sc_ = 9 followed by a linear increase at *N*_sc_ > 9. While the exact reason for this phenomenon is unknown, this study suggested that transitions in the scaling dependence and the onset of BB behavior begin to occur at very small *N*_sc_ values within the *n*_g_ = 1 limit.

Fewer dilute solution studies focus on less densely grafted BBs, such as PNBs, where *n*_g_ = 4. Verduzco and co-workers utilized SANS to study poly(oxanorbornene)-*g*-S BBs with varying *N*_bb_ (10–264) and *N*_sc_ (14–54) produced with the grafting-through method.^62^ Their findings agreed with similar studies performed on vinyl-based (*n*_g_ = 1) BBs where a more spherical geometry is realized at small *N*_bb_ (and relatively high *N*_sc_) that traverses to a more cylindrical structure as the *N*_bb_ ≫ *N*_sc_. Here, it should be noted that the BB chain ends, which are more abundant at lower *N*_bb_, allow the side-chains to extend more radially outward as a half-spherical “cap” at the ends. This helps paint a picture of the molecular shape traversing from spherical to “pill-shaped”, to cylindrical, as *N*_bb_ increases at a fixed *N*_sc_. A recent report by Sing and co-workers utilized a combination of computer simulations and experimentation to study poly(lactic acid), (PLA)-grafted NB BBs produced by the grafting-through method.^49^ Experimental materials had varying *N*_sc_ (30, 70, and a sweep of 18–103) and a large focus was placed on the effects of increasing *N*_bb_ to observe unique differences in conformational properties, asphericity, and prolateness imparted by these architectures. By increasing *N*_bb_ with a fixed *N*_sc_ or side-chain radius, the BBs traverse several molecular geometries (star-like, extended rod, and coil-like chains), and physical limits were identified where either the side-chain or the backbone dominates the molecular structure. Sunday et al. have also reported a combination of SANS and Monte Carlo simulations to elucidate molecular geometries of PNB(105)-*g*-S(40) as a function of solution concentration in a good solvent.^56^ They observed that BB conformations are highly sensitized to concentration, being more anisotropic at high dilutions and becoming increasingly isotropic at a higher concentration.

Despite these previously reported studies to elucidate dilute solution properties of BB systems, no report has ever utilized the grafting-from method to directly compare dilute solution scaling relationships of a backbone with fixed *N*_bb_ as a function of increasing *N*_sc_, particularly at lower (*N*_sc_ < 20) values. Furthermore, no study has been able to directly probe and compare two different backbone chemistries with identical graft densities (*n*_g_ = 4) as afforded by comparing PNB and PCP-based BB systems. Such a study will provide valuable insight into the onset of BB behavior by increasing *N*_sc_ at a fixed *N*_bb_ and is herein presented.

### Synthetic Discussion

While grafting-through is the most popular method for producing BBs from PNB derivatives, this method is less amenable for CP. Grafting-through of CP MMs is complicated by dilution of the polymerizable alkenes that result from preinstallation of the side chain. The equilibrium ROMP thermodynamics of low ring-strain monomers, such as CP, are sensitive to initial monomer concentration ([*M*]_0_), which deleteriously affects conversion if too diluted. NB has a higher ring-strain energy and is much more tolerant of this dilution; however, evidence has shown that enhanced sterics imposed by the grafts can effectively lower the ceiling temperature (*T*_c_) of these polymerizations, limiting *N*_bb_ to some extent and resulting in unreacted MMs that may be difficult to remove from the BB product. Grafting-from is void of complications arising from macromolecular contamination (i.e., unreacted MMs) and also provides a systematic approach to analyze increasing *N*_sc_ as a function of a well-defined, pre-characterized, *N*_bb_. For consistency and rigorous comparison, the grafting-from approach was performed on both PCPBIB and PNBBIB macroinitiators in this study. As a control, a PNB BB was also synthesized via grafting-through for direct comparison to the BBs produced by grafting-from and showed good agreement (Table S1).

Within the suite of materials synthesized for this study, we began with a series of linear PCPBIB (*N*_bb_ = 61–225) and PNBBIB (*N*_bb_ = 68–239) macroinitiators prepared via ROMP using varying monomer-to-initiator ratios ([*M*]_0_/[*I*]_0_) and analyzed them to determine scaling relationships of their dilute solution properties without grafts (*N*_sc_ = 0) (Table 1). Side chains were also prepared and measured independently by synthesizing a set of linear PS samples (*N*_n_ = 108–578) by ATRP using CPBIB as the initiating species.

From the set of macroinitiators, PCPBIB(61) and PCPBIB(111) as well as PNBBIB(68) and PNBBIB(122) were chosen for grafting-from studies. Their selection affords two comparative sets of BBs for each backbone with approximately double the *N*_bb_. ATRP of S performed at varying timestamps produced a range of *N*_sc_ ≈ 5–116 for each macroinitiator chosen (Tables 2 and 3). Our previous study showed that ATRP of S from PCPBIB produced near quantitative grafting efficiency, which was concluded by spectroscopic analysis and further confirmed by analyzing the linear PS grafts that were produced following quantitative RCMD of the backbone. The latter is a notable advantage afforded by leveraging the thermodynamics of polypentenamer-based BBs. As an added control in this study, we performed RCMD on PCPBIB(111)-*g*-S(33), PCPBIB(111)-*g*-S(60), and PCPBIB(111)-*g*-S(116) to produce CPBIB-PS(28), CPBIB-PS(55), and CPBIB-PS(73) (Table 1). The *N*_n_ of the depolymerized grafts showed good agreement with the *N*_sc_ calculated from the BB MW measured using SEC (Figure S17). However, the largest *N*_sc_ synthesized, CPBIB-PS(73), shows some deviation from the calculated BB graft length of *N*_sc_ = 116. Since this sample has the highest overall *M*_w_ (>1 million Da) and the largest side chains, we believe that the cause of this deviation may be 2-fold: Error in the SEC analysis due to the sample approaching or exceeding the column separation limits and error from ^1^H NMR analysis of the large grafts due to challenges of accurately integrating olefin signals within the confines of the signal-to-noise ratio and baseline resolution of the spectrum.

After synthesis of the PNBBIB-*g*-S BBs, a small, higher molar mass shoulder was seen on the SEC RI traces which we hypothesized to be from the propensity for PS to perform radical coupling and result in BB dimerization (Figures S14 and S15). Interestingly, this coupling reaction was not observed for the PCPBIB-*g*-S systems (Figure S12) under nearly identical conditions nor were they seen from the linear CP-PS SEC traces produced from RCMD of the BB or the linear CPBIB produced directly from ATRP (Figure S17). A variety of conditions were explored to prevent the higher molar mass shoulder in of PNB BBs in SEC RI traces including; reduced reaction temperature, less reactive ligands, cosolvents, increased dilution as well as higher equivalents of CuBr_2_. While complete prevention of coupling was unsuccessful, the best conditions found to minimize high molar mass shouldering were 700:1:0.4:0.05:0.8 for [S]_0_/[BIB]_0_/CuBr/CuBr_2_/PMDETA at 80 °C. For comparison of dilute solution properties, we also synthesized a PNB BB through a traditional grafting through method.^17^ The BB produced from grafting-through of NB macromonomers also displayed a higher molar mass shoulder in the SEC RI trace (Figure S16) which has also been seen in other studies and may be due to trace impurities caused by similar chain coupling during macromonomer synthesis.^82,83^ Since complete avoidance of the high molar mass shoulder could not be achieved, peak integration and calculation of molar mass, *D̵*, and other solution properties for the PNBBIB systems were performed to exclude this shoulder within the peak delimiters used. The properties measured for PNB BBs produced from the grafting-from and grafting-to methods were in good agreement. We anticipate that the PNB BBs produced by grafting-from also display near-quantitative grafting efficiency, even though they are not amenable to RCMD as was performed to confirm PCP BBs. Our confidence is bolstered by observing no residual PNBBIB backbone signals (Figure S5) in the ^1^H NMR spectra of the PNB BBs after grafting-from (Figure S8). Additionally, ^1^H NMR spectra of the grafting-from and grafting-through PNB BBs are identical. This indicates that near-quantitative initiation of PS grafts was achieved within the signal-to-noise limits of the NMR analysis. Another study that utilized ATRP grafting-from of PNB reported 95% grafting efficiency of a bulky monomer, *t*-butyl methacrylate when two ATRP handles per repeat unit were present.^84^ Their success, even when the steric impedance was high, provides further confidence in the robust nature of the grafting-from technique. Finally, as a means to explore the influence of graft chemistry, MA was grafted-from PCPBIB(61) with comparable *N*_sc_ ≈ 11–72 (Figure S18) to compare PCPBIB(61)-*g*-MA to the PCPBIB(61)-*g*-S series (Table 4).

### Intrinsic Viscosity

**Figure 2:**
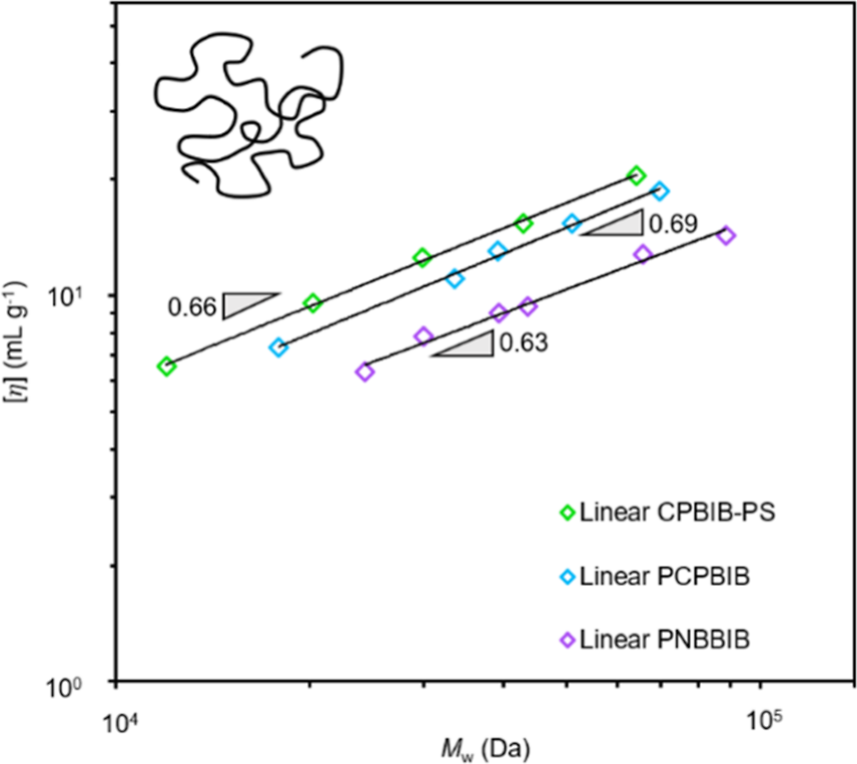
Intrinsic viscosity as a function of absolute molar mass for linear CPBIB-PS (green), PCPBIB (blue), and PNBBIB (purple) samples.

For the linear macroinitiators with *N*_sc_ = 0, [η] was plotted as a function of absolute molar mass (Figure 2) and fitted to a power-law to determine υ_iv_ (eq 1). A υ_iv_ value of 0.69, 0.63, and 0.66 was determined for PCPBIB, PNBBIB, and CPBIB-PS, respectively. An exponent of 0.6–0.8 for these linear analogs is consistent with a polymer coil in a good solvent.^85^ The υ_iv_ for PNBBIB is also in good agreement with previously reported data (υ_iv_ = 0.67) for poly(5-norbornene-2-methylbenzoate) in chlorobenzene at 30 °C.^49^ The CPBIB-PS exponent is slightly less than reported in the literature, which we attribute to the low molar mass of the samples. As expected, the CPBIB-PS samples produced from RCMD showed good agreement with the linear υ_iv_ scaling of the PS grown by ATRP (Figure S19). PCPBIB has a υ_iv_ that is 0.06 higher than that of PNBBIB. Although a minor difference, this slight increase may be due to the enhanced flexibility of PCPBIB and its ability to expand in solution.

**Figure 3:**
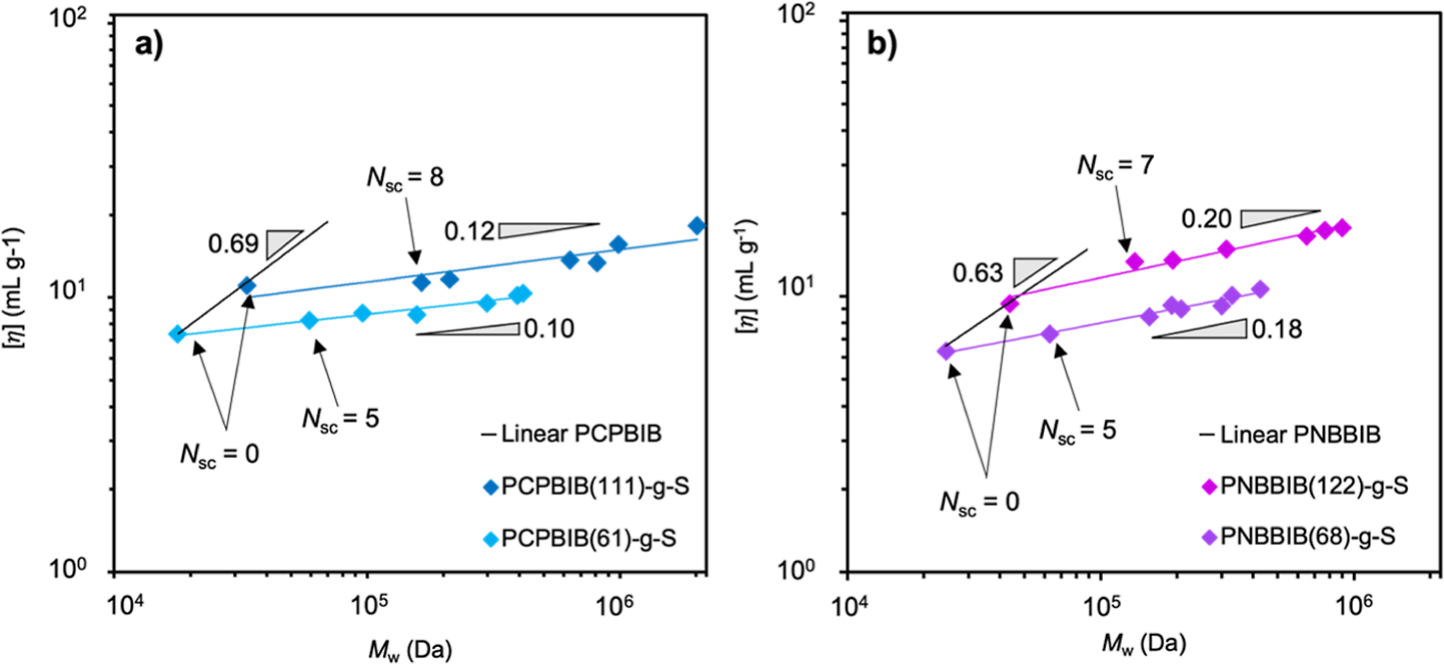
Overlaid plots of intrinsic viscosity as a function of absolute molar mass for (a) PCPBIB(61)-*g*-S (light blue) and PCPBIB(111)-*g*-S (dark blue) and (b) PNBBIB(68)-*g*-S (purple) and PNBBIB(122)-*g*-S (magenta). Black lines represent the slope of the respective linear macroinitiators (*N*_sc_ = 0) while the colored lines represent the linear regression of the data sets. Slopes (gray triangles) are presented as a guide to the eye.

Next, [η] was measured for all BB samples and plotted as a function of the absolute molar mass (Figure 3). For each BB set, the *M*_w_ of BBs increases solely as a function of increasing *N*_sc_, a unique perspective achieved by this study. An immediate and significant reduction in υ_iv_ is observed for both PNB- and PCP-based BBs when compared to their linear backbones. The onset of this reduced scaling begins with the lowest *N*_sc_ values of 5–11 and continues linearly throughout the samples with *N*_sc_ ≥ 60. Furthermore, it is observed that υ_iv_ is independent of BB backbone length yet slightly dependent on BB backbone chemistry. For example, both sets of the PCPBIB-*g*-S scale at υ_iv_ = 0.11 ± 0.01 while both sets of the PNBBIB-*g*-S scale at nearly double the exponent, υ_iv_ = 0.19 ± 0.01 (Figure 3). While the overall *M*_w_ spans two orders of magnitude for these BBs, only a slight increase in [η] is observed. This is consistent with previous reports on PNB- and poly(α-olefin)-based BBs and explained by the “stiffening” of the backbone as *N*_sc_ increases and occupies excluded volume in its proximity.^49,60^ However, the onset of this stiffening at *n*_g_ = 4 from very small *N*_sc_ (5–10) is a notable discovery of this work and suggests that only oligomeric side chains are necessary to achieve BB properties at moderate *N*_bb_. Since *n*_g_ and graft chemistry (in this case PS) are identical for these BBs and *N*_bb_/*N*_sc_ are closely matched, these slight variations in scaling can be directly attributed to the differences in repeating unit chemistry of the backbones (i.e., PCP vs PNB). One possible explanation for the lower scaling exponent of PCP BBs relative to that of PNB BBs is the bulkiness of the backbone chemistry (Figure 1). For the PNB BBs, a fused-maleimide ring occupies the immediate volume surrounding the backbone, causing the grafts to extend further away which affords a more expanded structure. The PCP backbone, however, is less congested in the absence of these rings and only boasts an ester group connecting the side chains. The grafts on the PCP backbone can therefore occupy more excluded volume near the backbone. Since [η] is inversely proportional to molecular density, a more expanded structure leads to a faster increase of [η] and a higher scaling for PNB BBs. While this is one hypothesis, it is expected that the bulkiness of the backbone repeat unit would have a less pronounced effect in the limit of high *N*_sc_; however, we see consistent scaling observations even for our longest synthesized side chain, which approach or exceed *N*_bb._ Another possible explanation can be derived from the theoretical efforts of Sing and co-workers where they reported that the Kuhn length (λ^–1^) increases with increasing *N*_sc_ (11–300) and that the observed scaling values also increased with increasing *n*_g_.^86^ As *N*_sc_ becomes larger, λ^–1^ could increase differently for the two backbone chemistries due to their intrinsic differences in flexibility, which may also cause differences in the observed scaling values. More experiments relating to quantifying λ^–1^ as a function of *N*_sc_ would provide a further understanding of the interactions that are occurring.

**Figure 4:**
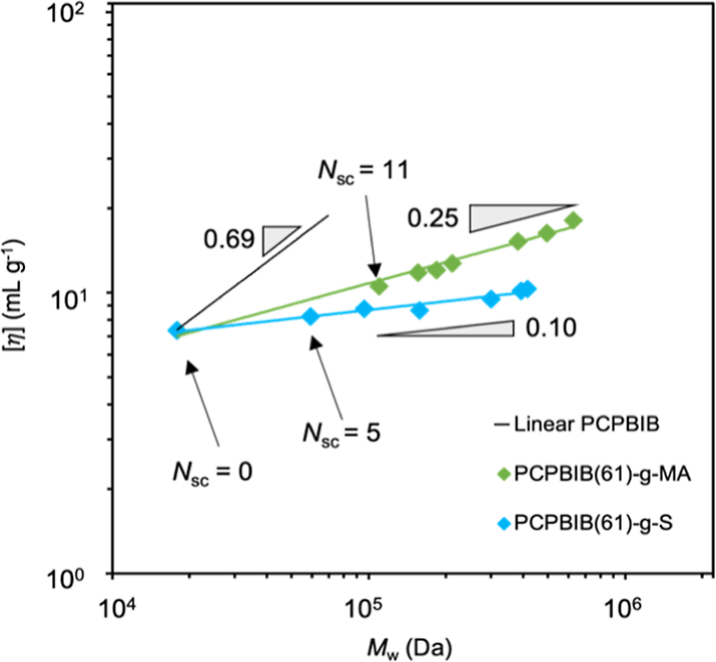
Overlaid plots of intrinsic viscosity as a function of absolute molar mass for PCPBIB(61)-*g*-S (blue diamonds) and PCPBIB(61)-*g*-MA (green diamonds). The black line represents the slope of the linear macroinitiators (*N*_sc_ = 0) while the colored lines represent the linear regression of the respective data sets. Slopes (gray triangles) are presented as a guide to the eye.

Another route of probing υ_iv_ is to fix all architectural parameters while only altering the side chain chemistry. To explore this, an additional set of BBs using PCPBIB(61) macroinitiator and grafting-from with (MA) was synthesized (Table 4) and [η] was plotted as a function of molar mass (Figure 4). Interestingly, PCPBIB(61)-*g*-MA displayed a much higher scaling relationship (υ_iv_ = 0.25) when compared to PCPBIB(61)-*g*-S side chains (υ_iv_ = 0.10). As expected, the side chain chemistry and its steric encumbrance are also significant factors that affect BB solution properties. In other words, the higher υ_iv_ of PCPBIB(61)-*g*-MA is caused by the bulkier MA side chains, imposing more steric hindrance and BB expansion.

In previous studies for PNB BBs, a small increase in υ_iv_ is seen as *N*_bb_ is increased (at a constant *N*_sc_) until a critical *N*_bb_ is reached where an uptick in υ_iv_ results in values similar to the respective linear backbones.^42,49^ While this study probes the opposite (i.e., constant *N*_bb_ and increasing *N*_sc_), deviations in scaling behavior are not observed for any BB samples in this study, even for samples where *N*_sc_ ≪ *N*_bb._ This again reinforces the claim that even oligomeric *N*_sc_ is suitable to produce BB behavior at a moderate *N*_bb_ (61–122).

### Hydrodynamic Radius

**Figure 5:**
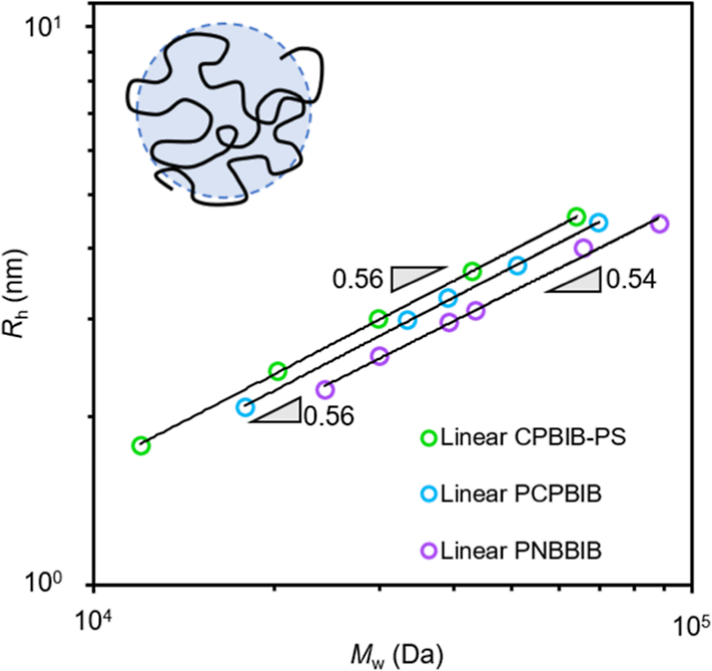
Hydrodynamic radius as a function of absolute molar mass for linear CPBIB-PS (green), PCPBIB (blue), and PNBBIB (purple) samples. Solid lines represent the linear fit of the respective data. Slopes (gray triangles) are presented as a guide to the eye.

When plotting *R*_h_ as a function of absolute molar mass, the *R*_h_ scaling relation, υ_R_h__ (eq 2) for linear PCPBIB, PNBBIB, and CPBIB-PS produce similar values of 0.56, 0.54, and 0.56, respectively (Figure 5).
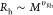
2

**Figure 6:**
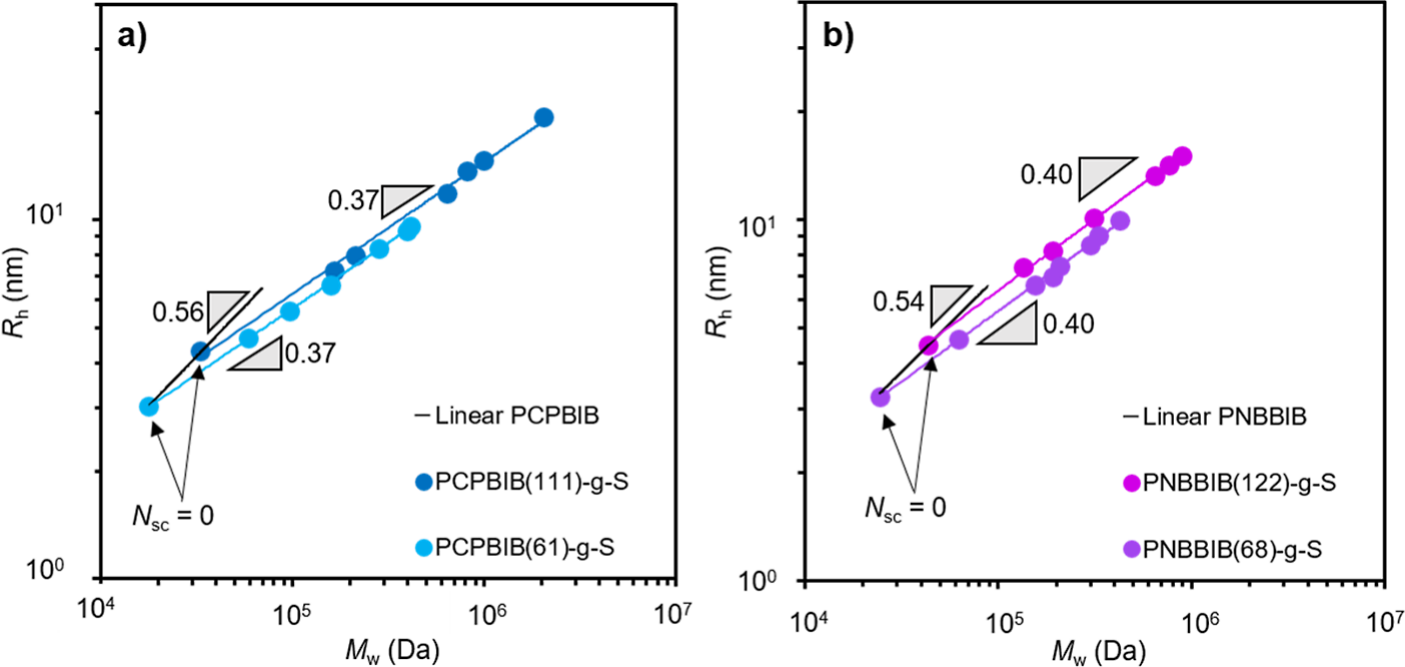
Overlaid plots of the hydrodynamic radius as a function of absolute molar mass for (a) PCPBIB(61)-*g*-S (light blue) and PCPBIB(111)-*g*-S (dark blue) and (b) PNBBIB(68)-*g*-S (purple) and PNBBIB(122)-*g*-S (magenta). Black lines represent the slope of the respective linear macroinitiators (*N*_sc_ = 0) while the colored lines represent the linear regression of the respective data sets. Slopes (gray triangles) are presented as a guide to the eye.

These values are also in good agreement with the Flory scaling exponent for an ideal, swollen linear chain (υ_R_h__ ≅ 0.59).^85^ Similar to [η], the *R*_h_ scaling exponent for all BBs is reduced compared to that of the corresponding linear backbone (Figure 6). Both sets of PCPBIB-*g*-S BBs scale with *M*_w_ such that υ_R_h__ = 0.37 while both sets of PNBBIB-*g*-S scale with υ_R_h__ = 0.40. These data show that, for PS side chains, increasing *N*_sc_ has the same effect on the overall size of the BB regardless of *N*_bb_. The reduction in υ_R_h__ from linear to BB yet consistency in υ_R_h__ for varied *N*_bb_ reiterates that increasing *N*_sc_ contributes less to the overall BB size compared to increasing *N*_bb,_ which has also been found in other studies.^49^

The slightly larger υ_R_h__ of the PNBBIB-*g*-S series compared to PCPBIB-*g*-S (0.37 vs 0.40, respectively) supports the previous conclusion that the PNB backbone is more congested, leading to a faster BB expansion rate in the presence of side chains which are unable to occupy excluded volume close to the backbone. It was anticipated that the difference in these two *R*_h_ scaling dependencies would be larger, similar to the large difference in [η] scaling for PCP and PNB BBs. However, *R*_h_ is reported to be less sensitive than [η] to small molecular modifications.^49^ This trend is also seen for PCPBIB(61)-*g*-MA, which exhibits a similar υ_R_h__ = 0.41 to that of the PCPBIB-*g*-S even though υ_iv_ was nearly doubled (Figure S20). Thus, this data confirm other reports that more prominent distinctions in υ_iv_ can be seen for small microstructural changes, while *R*_h_ is less sensitive.

### Radius of Gyration

**Figure 7:**
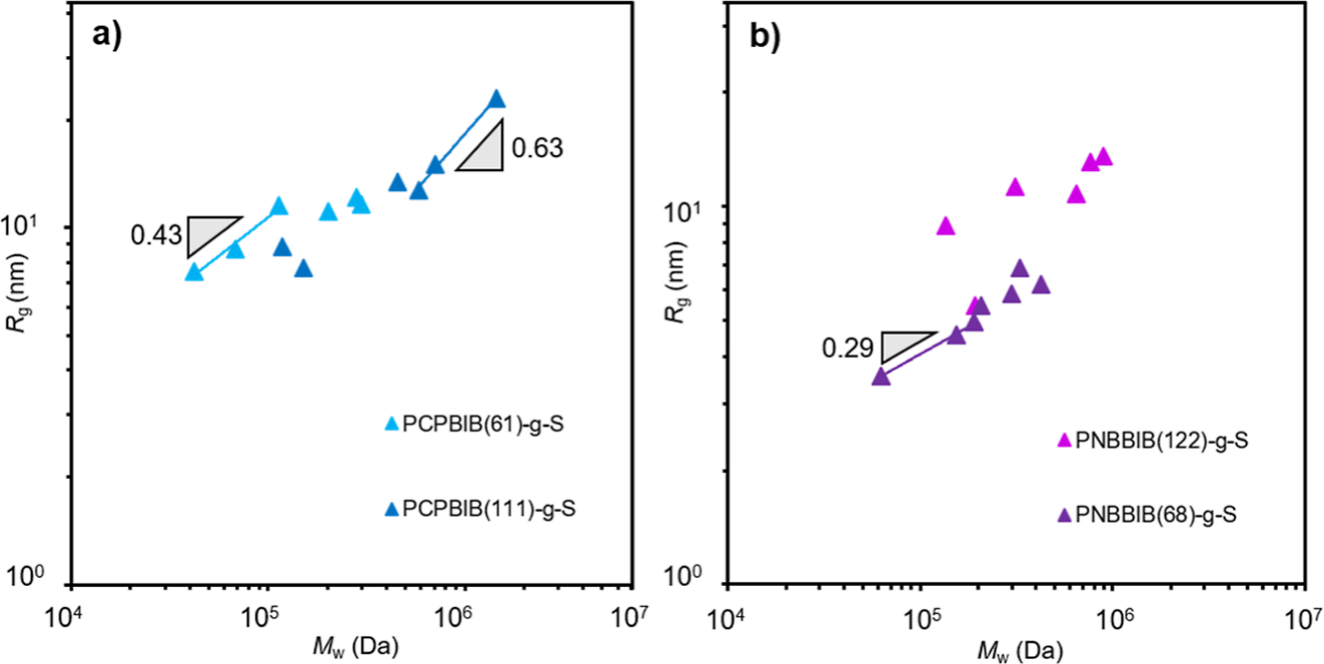
Overlaid plots of the radius of gyration as a function of absolute molar mass for (a) PCPBIB(61)-*g*-S (light blue) and PCPBIB(111)-*g*-S (dark blue) and (b) PNBBIB(68)-*g*-S (purple) and PNBBIB(122)-*g*-S (magenta). Colored lines represent the select linear fits across chosen data regions. Slopes (gray triangles) are presented as a guide to the eye.

The radius of gyration provides structural information and mass average distances between various molecular components. In general, the scaling values for *R*_g_ observed for each set of BBs did not trend linearly as was seen for [η] and *R*_h_, with the latter being a more dynamic representation. The nonlinear scaling likely results from the ensemble of *R*_g_ values extracted from many different possible conformations of a nonideal (low-to-moderate dispersity) sample.^87^ However, general trends can be elucidated by plotting *R*_g_ as a function of *M*_w_ (Figure 7). The values of *R*_g_ are close in magnitude to *R*_h_ with most being ∼8 ± 3 nm at intermediate *M*_w._ Additionally, a general increase in *R*_g_ occurs with increasing *N*_sc_ which is consistent with *R*_h_ data and with previous literature.^49,60^ Attempts to find linear trends and power exponents are shown (Figure 7) however, due to deviations in the data set, these are not to be highly interpreted. A better means to elucidate information from the *R*_g_ data is to take the ratio of *R*_g_/*R*_h_, known as the shape factor (*p*) which is provided for each BB set in Tables 2–4. The use of *p* allows the extraction of shape information about a macromolecule. For spherical geometries, *p* ≈ 0.78 and this value is expected to increase as the geometry becomes more cylindrical.^88^ When observing the trends of *p* for each BB set, it is clear that for lower *N*_sc_, *p* values are consistently higher and trend smaller as *N*_sc_ is increased. We interpret this to reflect the polymers adopting a nonspherical, more elongated cylinder structure when *N*_bb_ ≫ *N*_sc_, which is expected. For example, PCPBIB(61)-*g*-S(5) with small *N*_sc_ has *p* = 2.3 while PCPBIB(61)-*g*-S(49) has a reduced *p* = 1.7. Here, we note that deviations in the *R*_g_ values (Figure 7) are harbored within the deviations seen in some of the *p* values. It is also interesting to note that for PCPBIB(61)-*g*-S and PCPBIB(61)-*g*-MA, they encompass similar values of *p* as *N*_sc_ increases. This suggests that for a given backbone length of the same chemical composition, the BBs adopt similar shapes in their respective spherical-to-cylindrical transitions. The *p* values for PNBBIB-*g*-S, on the other hand, are more consistent in the spherical region of the shape spectrum, which is likely due to the different flexibility of the backbone and could contribute to the differences in observed scaling seen for more sensitive parameters like [η]. Other well-established techniques, such as SANS, could be used to better measure the absolute *R*_g_ values to provide further insight into the BB conformation.

## Conclusions

In this study, five sets of BBs of varying *N*_sc_ and *N*_bb_ were synthesized via grafting-from to probe differences in dilute solution as a function of *N*_sc_. Due to our prior success in expanding the limited synthetic variety of backbone chemistries, this study has uniquely allowed the direct comparison of two different backbones (PCP and PNB) with matched grafting density (*n*_g_ = 4) and probed the changes in their dilute solution properties as a function of increasing *N*_sc_ on varying but well-defined *N*_bb_. In addition, the solution properties of BBs comprising a polypentenamer backbone were analyzed with S and MA side chains for the first time. As anticipated, all BBs displayed a significant reduction in the scaling values of [η] and *R*_h_ when compared to that of linear polymer backbones (*N*_sc_ = 0) or side chains due to the dense grafting of the BBs. However, at the moderate *N*_bb_ values studied (61–122), a notable observation was that only oligomeric *N*_sc_ values (5–10) are necessary for the backbones to adopt BB characteristics in dilute solution, which is evidenced by a precipitous and linear reduction in the power law scaling of both [η] and *R*_h_ as a function of absolute molar mass. BBs containing the same backbone and side chain chemistry retain nearly identical scaling exponents for [η] and *R*_h_ regardless of *N*_bb_. However, [η] scales slightly higher for the more flexible polypentenamer-based BBs which also host grafts closer to their backbone. Other structural information, such as the shape factor (*p*), was also analyzed for each BB set as *N*_sc_ increased, and the general trends observed also support the evolution of a more elongated structure when *N*_bb_ ≫ *N*_sc_. This study adds a missing component to dilute solution property studies within the wealth of modifiable parameters for BB architecture and reiterates the significant effects they can have on the overall shape, size, and behavior of these macromolecules in solution. Future investigations are underway to probe how the polypentenamer backbone may affect the BB behavior in the melt state.

## Supporting Information Available

Materials purchased, synthetic methods, characterizations, and additional data such as NMR spectra, SEC traces, and additional dilute solution data (PDF)
