# Supplementary material for: Comparison of Polypentenamer and Polynorbornene Bottlebrushes in Dilute Solution
Source: ACS Polym Au. 2024 Feb 24;4(3):235–46. doi: 10.1021/acspolymersau.3c00052 (PMC11177302; doi:10.1021/acspolymersau.3c00052)
Supplement: Supplementary file 1 — lg3c00052_si_001.pdf [file lg3c00052_si_001.pdf]

Supporting Information

Comparison of Polypentenamer and Polynorbornene Bottlebrushes in Dilute Solution

Courtney M. Leo,[1] Jaehoon Jang,[1] Ethan J. Corey,[1] William J. Neary,[2] Jared I. Bowman,[3] Justin G. Kennemur*[1]

[1]Department of Chemistry and Biochemistry, Florida State University, Tallahassee, FL 32303, United States.

[2] Department of Chemistry, University of California at Riverside, Riverside, CA 92521, United States.

[3] George and Josephine Butler Polymer Research Laboratory, Center for Macromolecular Science & Engineering, Department of Chemistry, University of Florida, Gainesville, FL 32611, United States.

*E-mail: jkennemur@fsu.edu

Table of Contents

1. MATERIALS AND SYNTHETIC METHODS: 3
2. Materials. 3
3. Characterization. 3
4. Synthesis of cyclopent-3-en-1-yl-2-bromo-2-methylpropanoate (CPBIB): 3
5. Synthesis of poly(cyclopent-3-en-1-yl-2-bromo-2-methylpropanoate) (PCPBIB): 4
6. Synthesis of polystyrene using CPBIB as initiator (CPBIB-PS): 4
7. Grafting-from of polystyrene on PCPBIB (P(CPBIB-*g*-S)): 5
8. Grafting-from of poly(methyl acrylate) on PCPBIB (P(CPBIB-*g*-MA)): 5
9. Ring closing metathesis depolymerization (RCMD) of PCPBIB-*g*-S: 6
10. Synthesis of *exo*-norbornene anhydride (*exo*-NBA): 6
11. Synthesis of *exo*-*N*-(2-hydroxyethyl)-norbornene maleimide (NBOH): 7
12. Synthesis of *exo*-*N*-(2-((2-bromo-2-methylpropanoyl)oxy)ethyl)-norbornene maleimide (NBBIB) 7
13. Synthesis of Grubbs 3rd Generation Catalyst (G3) 8
14. Synthesis of poly[*exo*-*N*-(2-((2-bromo-2-methylpropanoyl)oxy)ethyl)-norbornene maleimide] (PNBBIB): 8
15. Synthesis of polystyrene initiated with NBBIB (NBBIB-PS) 9
16. Grafting-from of polystyrene on PNBBIB, (PNBBIB-*g*-S): 9
17. DATA AND CHARACTERIZATION 10

18. Figure S2. ^{1}H NMR (400 MHz, $CDCl_3$, 25 °C) of PCPBIB(61)-*g*-MA(17). 10
19. Figure S1. ^{1}H NMR (400 MHz, $CDCl_3$, 25 °C) of PCPBIB(111)-*g*-S(8). 10
20. Figure S3. ^{1}H NMR (400 MHz, $CDCl_3$, 25 °C) of NBBIB. 11
21. Figure S4. ^{13}C NMR (600 MHz, $CDCl_3$, 25 °C) of NBBIB. 11
22. Figure S5. ^{1}H NMR (400 MHz, $CDCl_3$, 25 °C) of PNBBIB(183). 12
23. Figure S6. ^{13}C NMR (600 MHz, $CDCl_3$, 25 °C) of PNBBIB. 12
24. Figure S7. ^{1}H NMR (400 MHz, $CDCl_3$, 25 °C) of NBBIB-PS(21). 13
25. Figure S8. ^{1}H NMR (400 MHz, $CDCl_3$, 25 °C) of PNBBIB(68)-*g*-S(23). 13
26. Figure S9. ^{1}H NMR (400 MHz, $CDCl_3$, 25 °C) of PNBBIB(56)-*g*-S(21) synthesized by grafting-through. 14
27. Figure S10. SEC RI traces of linear PCPBIB macroinitiators. PCPBIB(111) plotted separately due to analysis on a different SEC setup. 14
28. Figure S11. SEC RI traces of linear CPBIB-PS grafts. 15
29. Figure S12. SEC RI traces of PCPBIB(111)-*g*-S bottlebrush series. 15
30. Figure S13. SEC RI traces of linear PNBBIB macroinitiators. 16
31. Figure S14. SEC RI traces of PNBBIB(68)-*g*-S bottlebrush series. 16
32. Figure S15. SEC RI traces of PNBBIB(122)-*g*-S bottlebrush series. 17
33. Figure S16. SEC RI traces of NBBIB-PS(21) macromonomer and PNBBIB(56)-*g*-S(21) bottlebrush synthesized from the grafting-through method. 17
34. Figure S17. SEC RI traces of select PCPBIB(111)-g-S bottlebrushes (solid lines) and the respective linear CPBIB-PS grafts (dashed lines) produced after quantitative RCMD of the PCP backbone. The color of the solid line matches the color of the dashed line for the grafts produced... 18
35. Figure S18. SEC RI traces of PCPBIB(61)-*g*-MA bottlebrush series. 18
36. Figure S19. Intrinsic viscosity versus absolute molar mass for CPBIB-PS (open diamonds) grown directly by ATRP using CPBIB initiator and CPBIB-PS (solid diamonds) produced from RCMD of select bottlebrushes after ATRP grafting-from. 19
37. Figure S20. Hydrodynamic radius versus absolute molar mass for PCPBIB(61)-g-MA bottlebrushes. 20
38. Figure S21. Radius of gyration versus absolute molar mass for PCPBIB(61)-g-MA bottlebrush series (green triangles) and comparative overlay of the PCPBIB(61)-g-S series (blue triangles) 20
39. Table S1. Comparative characterization data for BB sample synthesized via grafting-through and other BB samples produced from grafting-from. 21
40. REFERENCES: 21

MATERIALS AND SYNTHETIC METHODS:

Materials. All materials were used as received unless otherwise specified. Activated basic alumina and ethyl vinyl ether (EVE) (99%) were purchased from Alfa Aesar and used as received. Acetic acid (glacial), α-bromoisobutyryl bromide (BIBB) (98%), copper bromide (CuBr) (99%), copper (II) bromide ($CuBr_2$) (98%), magnesium sulfate (≥99%), *N,N,N',N'',N''*-pentamethyldiethylenetriamine (PMDETA) (99%), styrene (S), triethylamine (TEA) (>99%), and 1st Generation Grubbs Catalyst® (G1) (Umicore M102) and 2nd Generation Grubbs Catalyst® (G2) (Umicore M204) were purchased from Sigma-Aldrich. Sodium carbonate (99.5%), hexanes, diethyl ether (DEE), ethanol (EtOH), and ethyl acetate (EtOAc) were purchased from VWR Chemicals (BDH). 3-Cyclopenten-1-ol (3CPOH) (98%) was purchased from PharmaBlock. toluene, dichloromethane (DCM), and tetrahydrofuran (THF) were obtained from an SG Waters glass contour solvent purification system that was packed with neutral alumina. The solvents were passed through a 2 μm filter before being dispensed. S and PMDETA inhibitors were removed by passage through basic alumina. CuBr was purified by from glacial acetic acid followed by three washes with EtOH and DEE and dried under high vacuum at 25 ºC.

Characterization. ^{1}H NMR and ^{13}C NMR experiments were performed on a Bruker Avance III 400 or 600 MHz spectrometer within the FSU Chemistry and Biochemistry NMR Facility. Polymer molar mass, *Đ*, [η], R_h, and R_g were determined via two different Agilent – Wyatt combination triple detection size exclusion chromatography (SEC) instrument as described in detail the main manuscript.

Synthesis of cyclopent-3-en-1-yl-2-bromo-2-methylpropanoate (CPBIB):

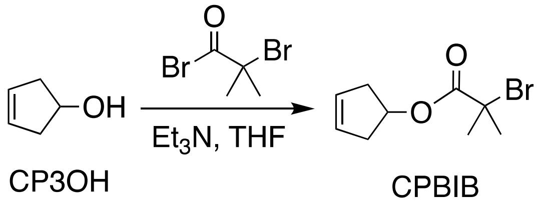

CPBIB monomer was synthesized as described in previous literature.[1] ^{1}H NMR (400 MHz, $CDCl_3$, 25 °C): δ (ppm) 5.72 (s, 2H), 5.43 (tt, J = 7.0, 2.5 Hz, 1H), 2.77 (dd, J = 16.9, 7.0 Hz, 2H), 2.49 – 2.39 (m, 2H), 1.95 (d, J = 15.7 Hz, 1H), 1.91 (s, 6H). ^{13}C NMR (600 MHz, $CDCl_3$, 25 °C): δ (ppm) 171.6, 128.2, 75.9, 56.2, 39.4, 30.7.

Synthesis of poly(cyclopent-3-en-1-yl-2-bromo-2-methylpropanoate) (PCPBIB):

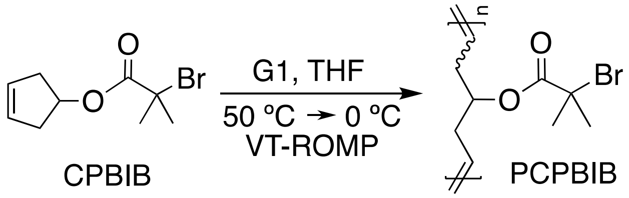

This synthesis is adapted from a previously reported procedure.[1] In a representative example; An oven-dried round bottom flask equipped with a magnetic stir bar, THF and CPBIB was added. The flask was capped with a septum and sparged with Ar. In a separate round bottom flask equipped with a magnetic stir bar, a predetermined mass of G1 was added and capped with a septum. The flask was gently purged for 5 min with argon before sparged THF was added. The catalyst solution was allowed to stir until dissolved. The two flasks containing the CPBIB and G1 solutions were equilibrated in an oil bath set to 50 ºC for 3 min. To the stirring CPBIB solution, the G1 solution was quickly injected via syringe. The total amount of THF upon combination with the catalyst injection resulted in an initial monomer concentration, $[M]_0 = 3.0$ M. The reaction was allowed to initiate at 50 ºC for 5 min. The solution was then transferred to a chiller at 0 ºC with rapid stirring and the ROMP proceeded for 5 h. At that time, excess EVE was added and stirred at 0 °C for 1 h to terminate the reaction. A small aliquot was taken for determination of monomer conversion by ^{1}H NMR. Equilibrium conversion was determined to be 63-84% in all cases. The remaining polymer solution was diluted with THF and passed through basic alumina to remove residual catalyst. The solution was then concentrated and precipitated into cold methanol. The polymer was collected and dried in a vacuum oven at r.t. to yield PCPBIB.

^{1}H NMR (400 MHz, $CDCl_3$, 25 °C) δ (ppm): 5.44 (br s, 2H), 4.87 (br s, 1H), 2.29 (br s, 4H), 1.89 (s, 6H). ^{13}C NMR (600 MHz, $CDCl_3$, 25 °C): δ (ppm) 171.6, 128.2, 75.9, 56.2, 39.4, 30.7. Consistent with previous literature.[1]

Synthesis of polystyrene using CPBIB as initiator (CPBIB-PS):

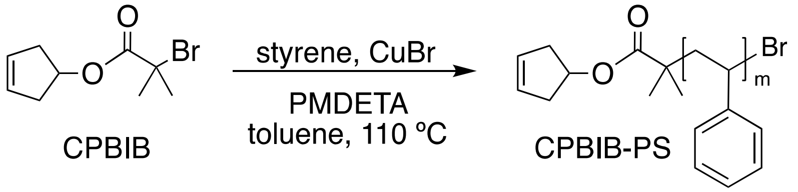

In a representative example, 50 μL (0.28 mmol) of CPBIB, 5.86 g (56.2 mmol, 200 eq.) of S, and 59 uL (0.28 mmol, 1 eq.) of PMDETA were added to toluene in a Schlenk flask with a magnetic stirrer. The solution was degassed through three freeze pump thaw (FPT) cycles. At that time, 40 mg of CuBr (28.1 mmol, 1 eq.) was added under Ar, resealed, and placed into a preheated oil bath at 110 °C. The solution was reacted for a specific timestamp depending on the molar mass desired followed by cooling in an ice bath, exposure to air, and dilution with DCM. The crude solution was passed through neutral alumina plug to remove copper catalyst. The polymer solution was then concentrated and precipitated in MeOH. The polymer was collected, redissolved, and

reprecipitated 2 additional times. The polymer was then collected and dried in a vacuum oven overnight.

^{1}H NMR (400 MHz, $CDCl_3$) δ 7.45 – 6.27 (m, 107H), 5.66 (s, 2H), 4.84 (d, J = 7.7 Hz, 0H), 4.47 (d, J = 6.5 Hz, 0H), 2.69 – 1.23 (m, 57H), 1.02 – 0.92 (m, 8H), 0.87 (s, 2H).

Grafting-from of polystyrene on PCPBIB (P(CPBIB-*g*-S)):

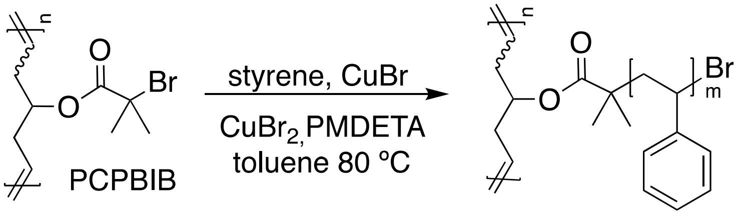

P(CPBIB-*g*-S)

This procedure is adapted from a previously reported procedure.[1] In a dry Schlenk flask equipped with a magnetic stir bar, a 50 mg mL^{-1} solution of PCPBIB and toluene was prepared and allowed to stir. After several minutes, styrene (600 eq.), PMDETA (0.8 eq.), and $CuBr_2$ (0.03 equiv.) was added and degassed with 3 freeze-pump-thaw cycles. Once complete, CuBr (0.4 eq.) was added under a positive nitrogen flow. The flask was resealed and placed into a preheated oil bath at 80 ºC. Sample aliquots were taken at various timestamps as N_{sc} increased. Each aliquot was cooled in an ice bath, opened to air, diluted, then passed through basic alumina to remove residual copper. Volatiles, including excess monomer were removed in *vacuo*. Remaining solids were dissolved in DCM and precipitated into cold MeOH. The dissolution/precipitation procedure was performed twice before the final pure polymer was collected and dried in a vacuum oven.

^{1}H NMR (400 MHz, $CDCl_3$) δ 7.42 – 6.23 (m, 171H), 5.08 (s, 2H), 4.42 (s, 2H), 1.49 (d, J = 20.0 Hz, 106H), 0.84 (d, J = 47.4 Hz, 7H).

Grafting-from of poly(methyl acrylate) on PCPBIB (P(CPBIB-*g*-MA)):

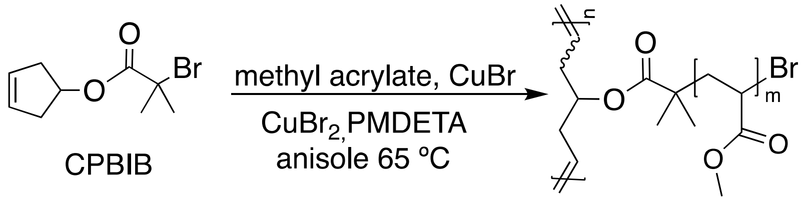

P(CPBIB-*g*-MA)

In a dry Schlenk flask equipped with a magnetic stir bar, a solution of PCPBIB in anisole (30 mg mL^{-1}) was prepared. Methyl acrylate (200 eq.), PMDETA (0.5 eq.) $CuBr_2$ (0.03 eq) were added and the homogeneous solution was degassed with 3 freeze-pump-thaw cycles. Under a positive N_2 blanket, CuBr (0.5 eq.) was added. The flask was resealed and placed into a preheated oil bath at

65 ºC. Aliquots were taken at various timestamps as N_{sc} increased. Each aliquot was cooled in an ice bath, opened to air, diluted, then passed through basic alumina to remove copper. Volatiles, including excess monomer were removed in *vacuo*. Remaining solids were dissolved in DCM and precipitated into cold MeOH. The dissolution/precipitation procedure was performed twice before the final pure polymer was collected and dried in a vacuum oven.

^{1}H NMR (600 MHz, $CDCl_3$) δ 5.31 (d, *J* = 24.3 Hz, 2H), 4.73 (s, 1H), 4.23 (dq, *J* = 6.4, 4.1 Hz, 1H), 3.77 (s, 4H), 3.65 (s, 114H), 2.29 (s, 43H), 2.04 – 1.83 (m, 19H), 1.69 (d, *J* = 24.6 Hz, 40H), 1.60 – 1.37 (m, 20H), 1.06 (d, *J* = 11.6 Hz, 6H).

Ring closing metathesis depolymerization (RCMD) of PCPBIB-*g*-S:

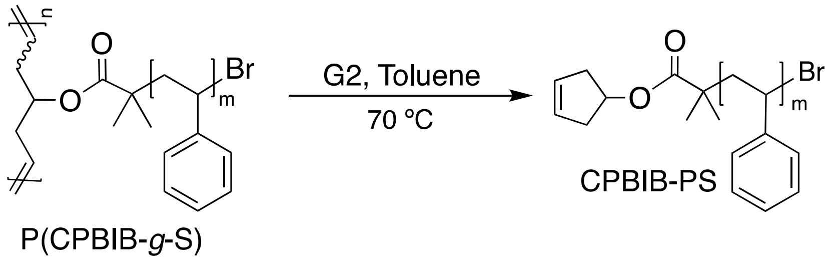

This procedure is adapted from a previously reported procedure.[1] To a dry flask, 0.1 g of PCPBIB-*g*-S was added and capped with a septum. The flask was purged with Ar for 5 min before dry, Ar-sparged toluene was added and the polymer was completely dissolved under stirring. A G2 catalyst solution was made separately at a known concentration in degassed toluene. A predetermined quantity of catalyst solution was injected into the PCPBIB-*g*-S solution. After this injection the initial solution concentration of olefins $[olefin]_0$ was 30 mM and the catalyst was at a 5 mol% catalyst loading relative to $[olefin]_0$. The solution was stirred at 70 ºC for 2 h. The reaction was quenched with excess EVE and the depolymerized grafts were then precipitated into MeOH and dried *in vacuo.*

^{1}H NMR (400 MHz, $CDCl_3$) δ 7.54 – 6.27 (m, 182H), 5.66 (s, 2H), 4.84 (d, *J* = 7.7 Hz, 0H), 4.47 (d, *J* = 6.5 Hz, 0H), 3.13 – 1.20 (m, 62H), 1.03 – 0.92 (m, 7H), 0.87 (s, 2H).

Synthesis of *exo*-norbornene anhydride (*exo*-NBA):

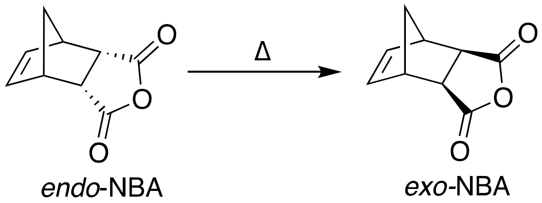

exo-Norbornene anhydride was prepared according to a previous procedure.[2]

^{1}H NMR (400 MHz, $CDCl_3$, 25 °C): δ (ppm) 6.35 – 6.31 (m, 2H), 3.45 (tp, J = 4.2, 2.0 Hz, 2H), 3.00 (d, J = 2.1 Hz, 2H), 1.69 – 1.63 (m, 1H), 1.44 (dd, J = 10.5, 5.2 Hz, 1H). ^{13}C NMR (600 MHz, $CDCl_3$, 25 °C): δ 171.3, 135.5, 52.8, 47.1, 46.1.

Synthesis of *exo*-*N*-(2-hydroxyethyl)-norbornene maleimide (NBOH):

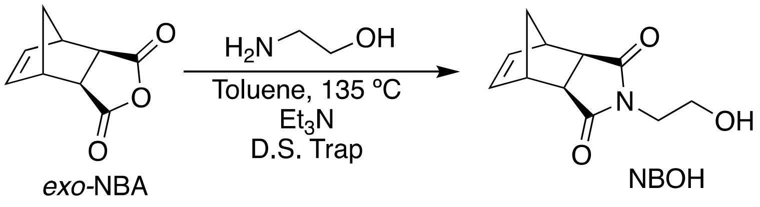

NBOH was prepared by previously reported procedures.[3,4]

^{1}H NMR (400 MHz, $CDCl_3$, 25 °C): δ (ppm) 6.28 (t, J = 1.9 Hz, 2H), 3.76 (dd, J = 5.8, 2.4 Hz, 2H), 3.69 (tt, J = 4.8, 2.7 Hz, 2H), 3.27 (p, J = 1.8 Hz, 2H), 2.71 (q, J = 1.5 Hz, 2H), 2.33 (s, 1H), 1.55 – 1.46 (m, 1H), 1.37 – 1.30 (m, 1H). ^{13}C NMR (600 MHz, $CDCl_3$, 25 °C): δ (ppm): 178.8, 137.8, 60.6, 47.9, 45.3, 42.8, 41.4. Consistent with literature.[3,4]

Synthesis of *exo*-*N*-(2-((2-bromo-2-methylpropanoyl)oxy)ethyl)-norbornene maleimide (NBBIB):

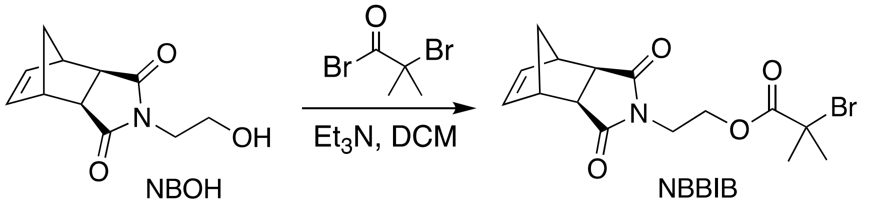

NBBIB was prepared by adapting a previously reported procedure.[1] To an oven dried round bottom flask equipped with a magnetic stir bar, NBOH (4.60 g, 22.2 mmol), TEA (3.17 mL, 26.6 mmol), and 30 mL of DCM was added under argon followed by cooling to 0 °C. A mixture of BIBB (3.30 mL, 26.6 mmol) in 30 mL of DCM was added to an addition funnel and added drop-wise. After complete addition, the reaction was stirred at 23±2 °C overnight. The solution was filtered and concentrated by rotary evaporation. A separatory funnel was used to wash the organic layer with successive washes of 0.1 M Na_2CO_3, distilled H_2O, and NaCl brine, respectively. The organic layer was then dried over $MgSO_4$, filtered, and volatiles were removed to yield a white solid (6.43g, 82% yield).

^{1}H NMR (400 MHz, $CDCl_3$, 25 °C): δ (ppm) 6.27 (t, J = 1.9 Hz, 1H), 4.31 (t, J = 5.1 Hz, 1H), 3.80 (t, J = 5.1 Hz, 1H), 3.28 – 3.24 (m, 1H), 2.69 (d, J = 1.5 Hz, 1H), 1.87 (s, 3H), 1.51 (dt, J = 10.0,

1.7 Hz, 1H), 1.30 (d, J = 9.9 Hz, 1H). ^{13}C NMR (600 MHz, $CDCl_3$, 25 °C): δ (ppm) 177.68, 137.81, 62.57, 55.47, 47.85, 45.19, 42.91, 37.34, 30.60.

Synthesis of Grubbs 3rd Generation Catalyst (G3)

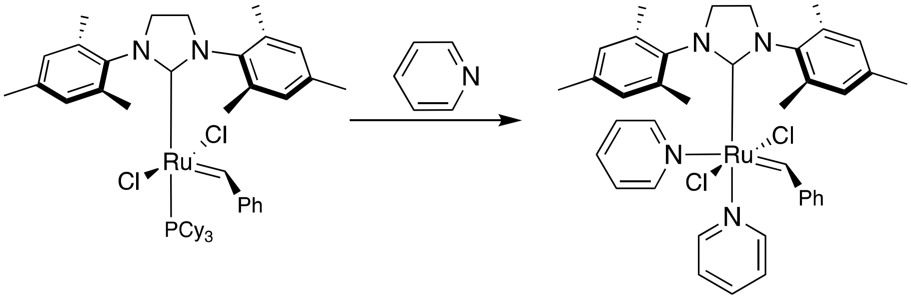

This synthesis was adapted from a previously reported procedure.[5] In a 20 mL scintillation vial, 0.150 g (0.177 mmol) of G2 was dissolved in excess pyridine (80 eq.) and stirred at 23±2 for 30 min. Hexanes was layered on top and the solution was placed in the freezer. The precipitate was collected by gravity filtration and washed thrice with excess cold hexane. The recovered solid was then dried under vacuum overnight resulting in a bright green powder (0.11 g, 83%).

Synthesis of poly[*exo-N*-(2-((2-bromo-2-methylpropanoyl)oxy)ethyl)-norbornene maleimide] (PNBBIB):

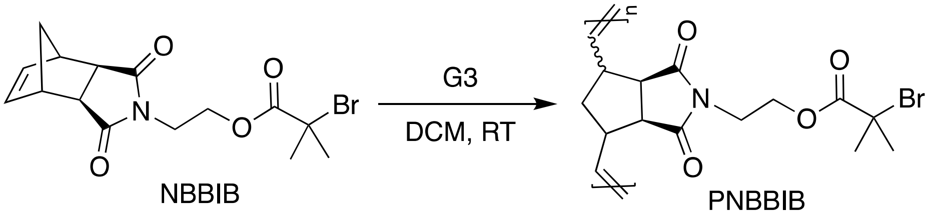

In a representative example, a dry flask with a magnetic stir bar, was charged with predetermined amount of NBBIB. The flask was capped with a septum and sparged with Ar for 5 min followed by addition of DCM that had been previously sparged with Ar. In a separate flask, equipped with a magnetic stir bar, a predetermined quantity of G3 was added and the flask was capped with a septum. The flask was then purged gently for 5 min with Ar before sparged DCM was added to dissolve the catalyst with stirring. To the stirring flask with NBBIB, the G3 solution was quickly injected via syringe. The amount of combined DCM after injection was predetermined to bring the initial monomer concentration to $[M]_0$ = 0.05 M. The mol % G3 injected was based on the desired N_{bb} for each reaction. The reaction was stirred for 1 h and the polymerization was then quenched with excess EVE. This solution was then stirred for a minimum of 1 h. A small aliquot of the crude ROMP solution was taken for ^{1}H NMR characterization to determine monomer conversion which was near quantitative in all cases. The remaining solution was diluted with DCM and passed through basic alumina to remove catalyst. The solution was filtered, concentrated, and precipitated

into cold methanol. The dissolution/precipitation procedure was performed twice before the final pure polymer was collected and dried in a vacuum oven.

Synthesis of polystyrene initiated with NBBIB (NBBIB-PS)

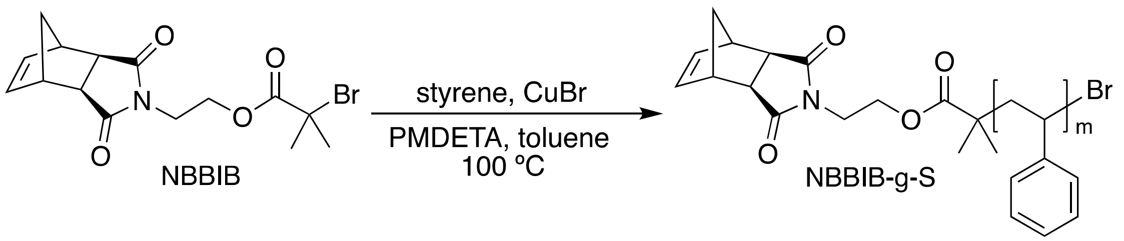

The procedure for synthesizing NBBIB-PS was identical to the procedure described above for the synthesis of CPBIB-PS with the exception that these polymerizations were performed at 100 °C.

^{1}H NMR (600 MHz, $CDCl_3$) δ 7.41 – 6.30 (m, 102H), 6.26 (s, 2H), 4.42 (s, 1H), 3.65 – 3.29 (m, 4H), 3.22 (s, 2H), 2.62 (d, J = 9.1 Hz, 2H), 2.59 – 1.26 (m, 64H), 1.18 (s, 1H), 0.88 (q, J = 21.2 Hz, 7H).

Grafting-from of polystyrene on PNBBIB, (PNBBIB-*g*-S):

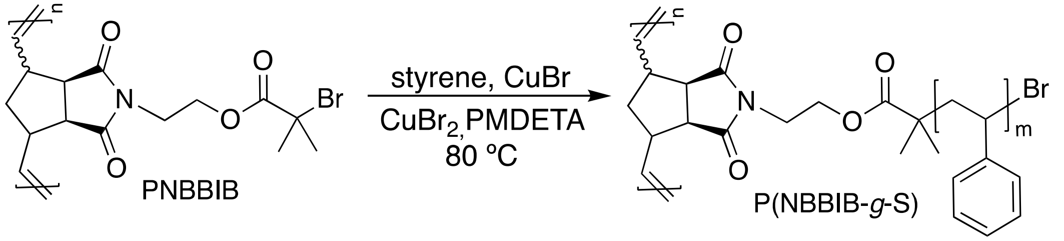

This procedure is adapted from a previously reported procedure.[1] In a dry Schlenk flask equipped with a magnetic stir bar, PNBBIB (mol BIB = 1 eq.), styrene (700 eq.), PMDETA (0.8 eq.), and $CuBr_2$ (0.05 eq) was added and degassed with 3 freeze-pump-thaw cycles. Under a positive N_2 blanket, CuBr (0.4 eq.) was added. The flask was resealed and placed into a preheated oil bath at 80 ºC. Aliquots were taken at various timestamps as N_{sc} increased. Each aliquot was cooled in an ice bath, opened to air, diluted, then passed through basic alumina to remove copper. Volatiles, including excess monomer, were removed in *vacuo*. Remaining solids were dissolved in DCM and precipitated into cold MeOH. The dissolution/precipitation procedure was performed twice before the final pure polymer was collected and dried in a vacuum oven.

^{1}H NMR (400 MHz, $CDCl_3$) δ 7.8 – 6.25 (br, aryl-H), 5.75–5.25 (br, olefin-H), 4.7–1.3 (br, PS methine-H), 3.7 – 1.25 (br, m, various sp^3-H signals, see Figure S8), 1.2–075 (br, methyl-H).

DATA AND CHARACTERIZATION :

Full characterization data for PCPBIB(61)-g-S series is found in previously literature. [1] To ensure accuracy and consistency with the analyses of all samples in this manuscript, the data from those samples was reanalyzed using consistent integration procedures. As a result, minor deviations in MW and N_n of the samples is seen.

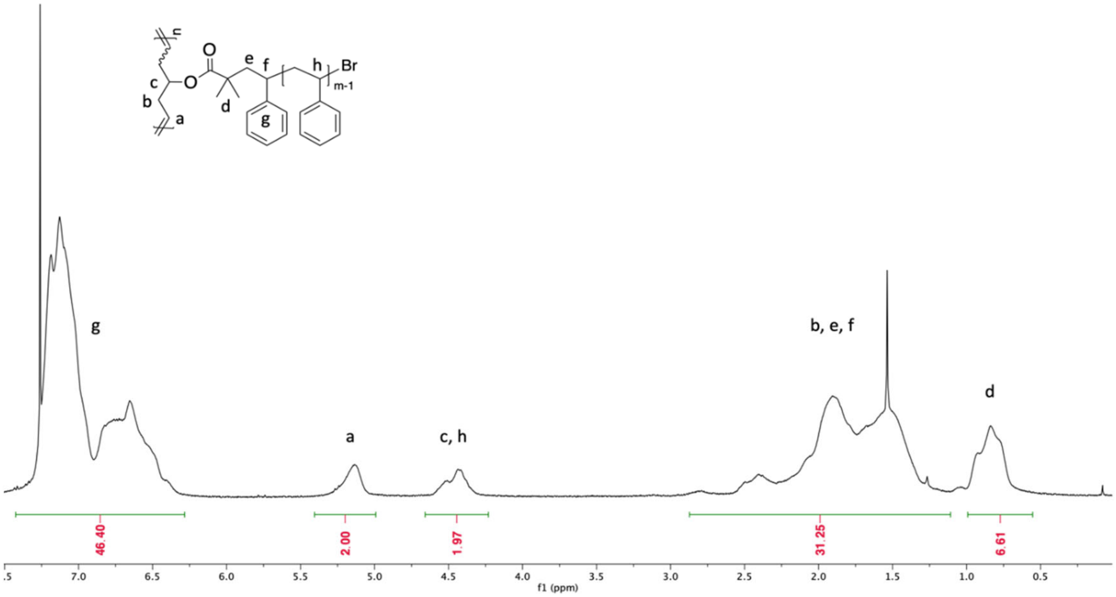

Figure S1. ^{1}H NMR (400 MHz, $CDCl_3$, 25 °C) of PCPBIB(111)-*g*-S(8).

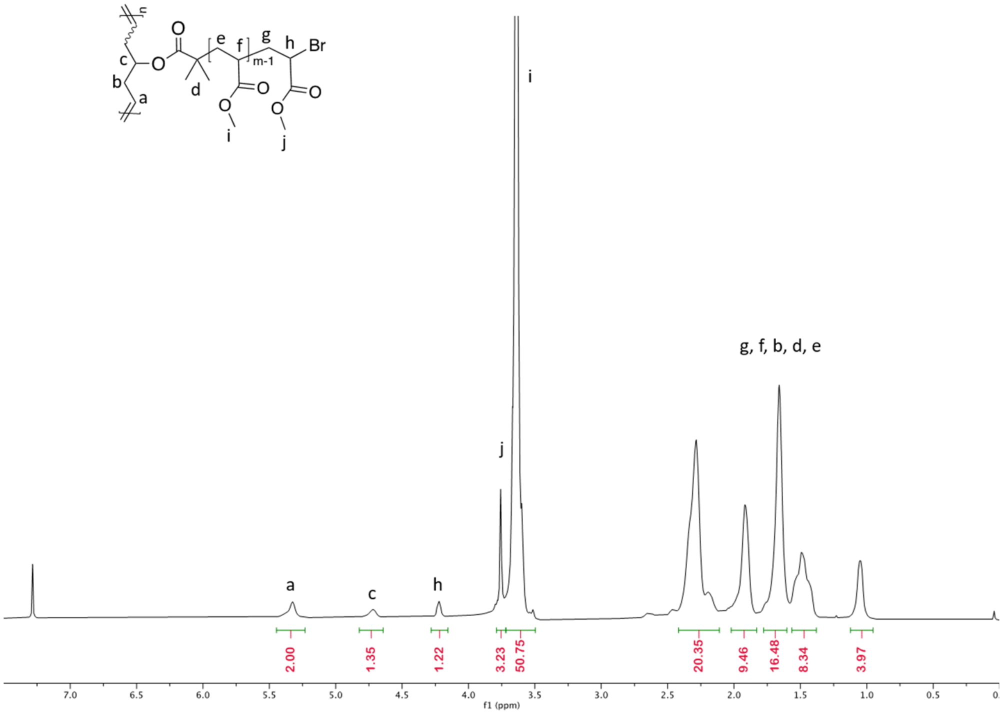

Figure S2. ^{1}H NMR (400 MHz, $CDCl_3$, 25 °C) of PCPBIB(61)-*g*-MA(17).

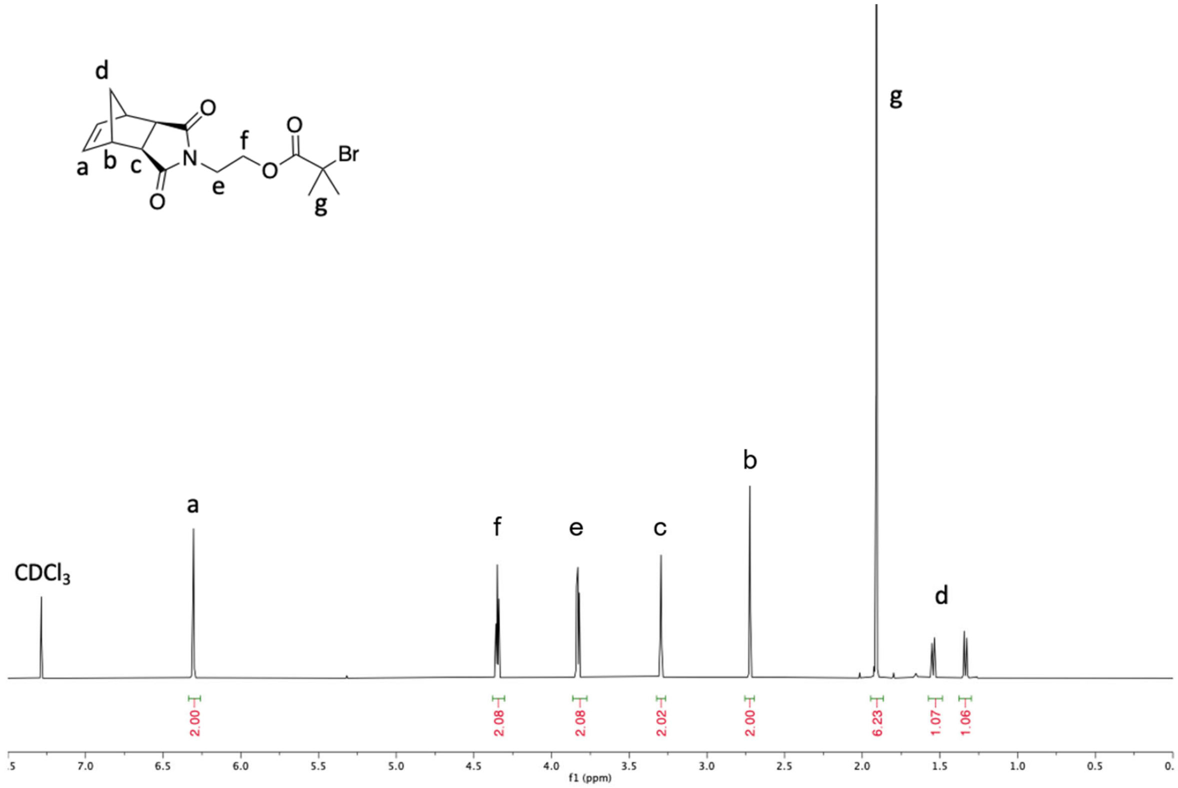

Figure S3. ^{1}H NMR (400 MHz, $CDCl_3$, 25 °C) of NBBIB.

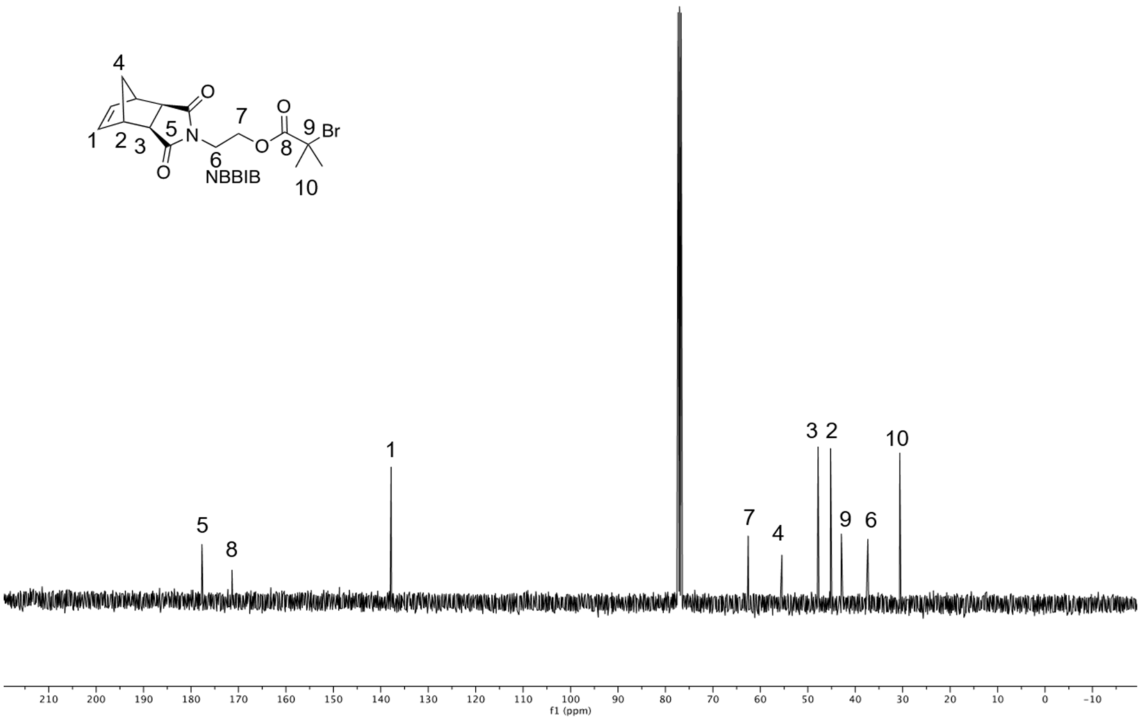

Figure S4. ^{13}C NMR (600 MHz, $CDCl_3$, 25 °C) of NBBIB.

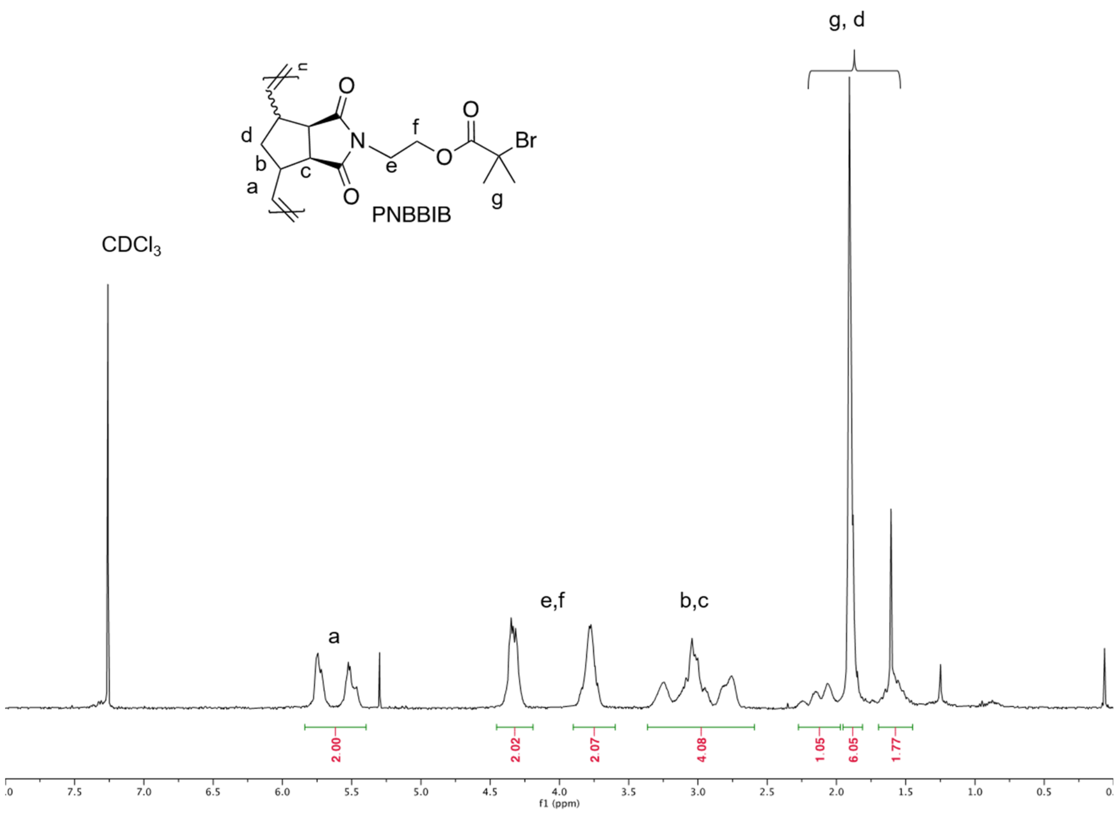

Figure S5. ^{1}H NMR (400 MHz, $CDCl_3$, 25 °C) of PNBBIB(183).

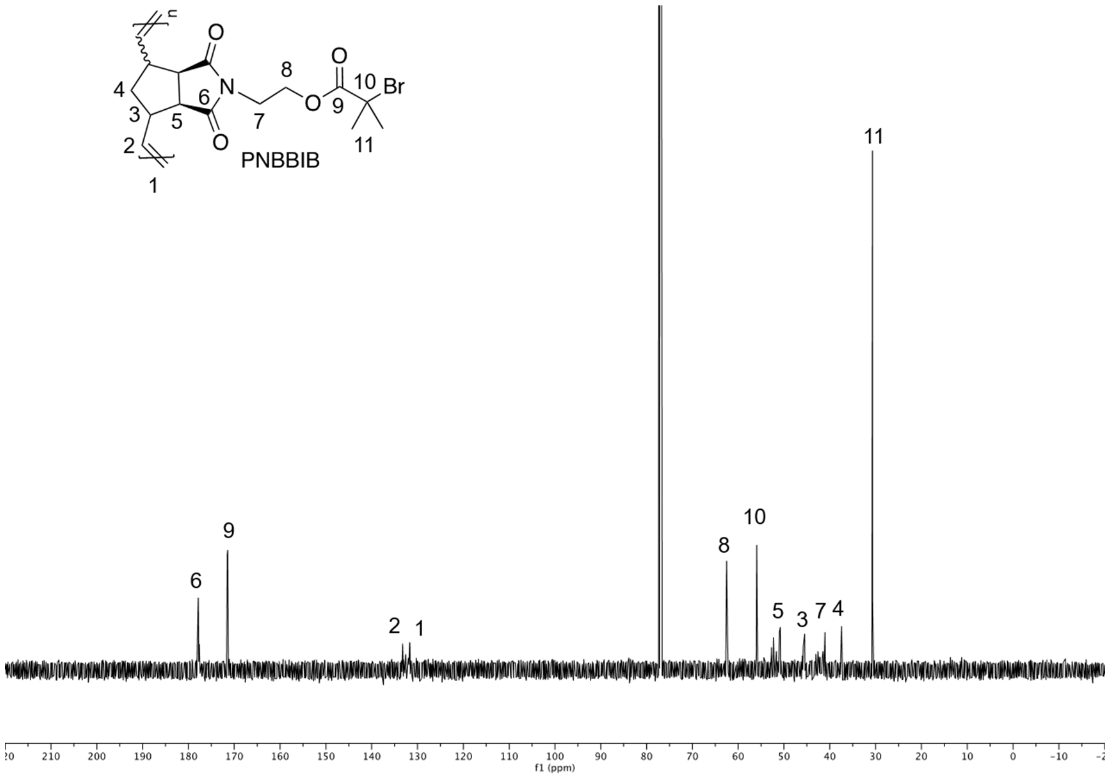

Figure S6. ^{13}C NMR (600 MHz, $CDCl_3$, 25 °C) of PNBBIB**.**

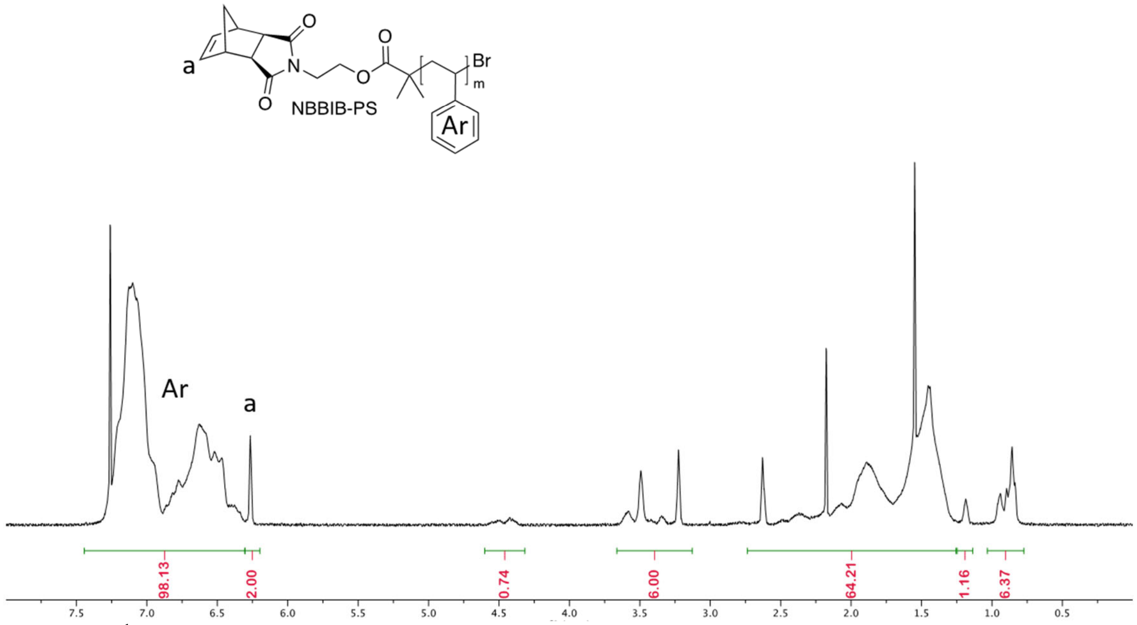

Figure S7. ^{1}H NMR (400 MHz, $CDCl_3$, 25 °C) of NBBIB-PS(21).

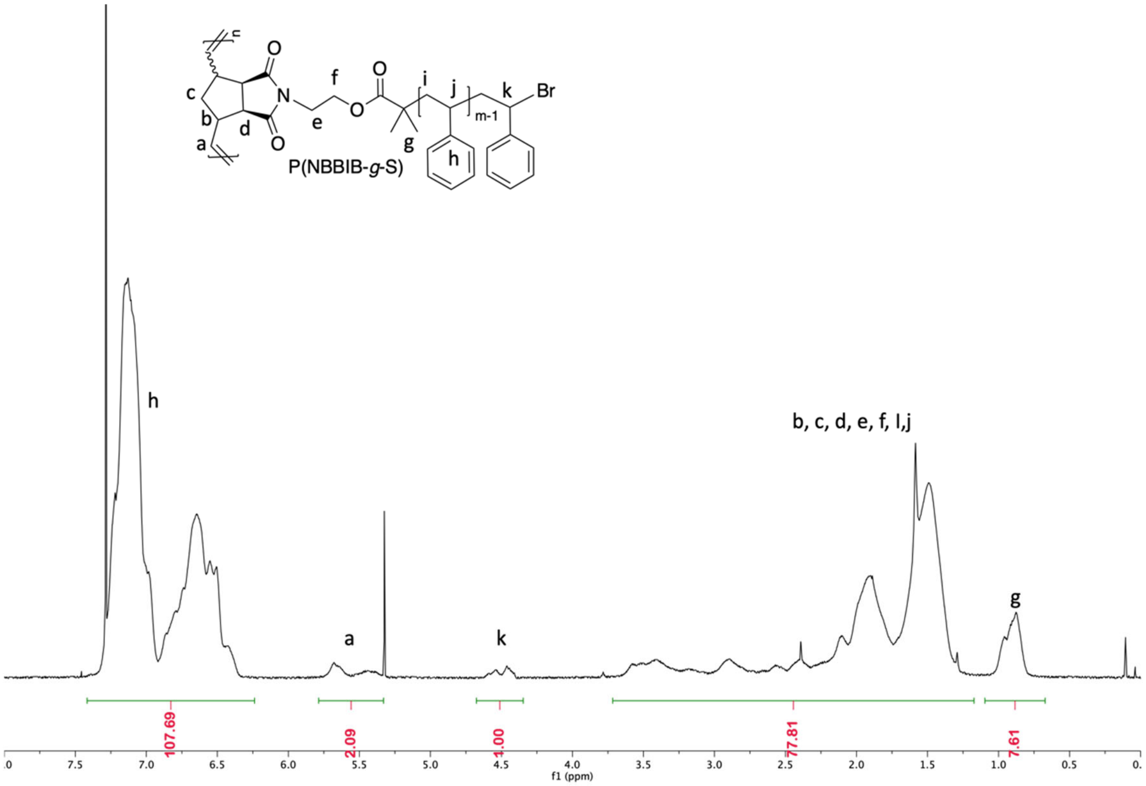

Figure S8. ^{1}H NMR (400 MHz, $CDCl_3$, 25 °C) of PNBBIB(68)-*g*-S(23).

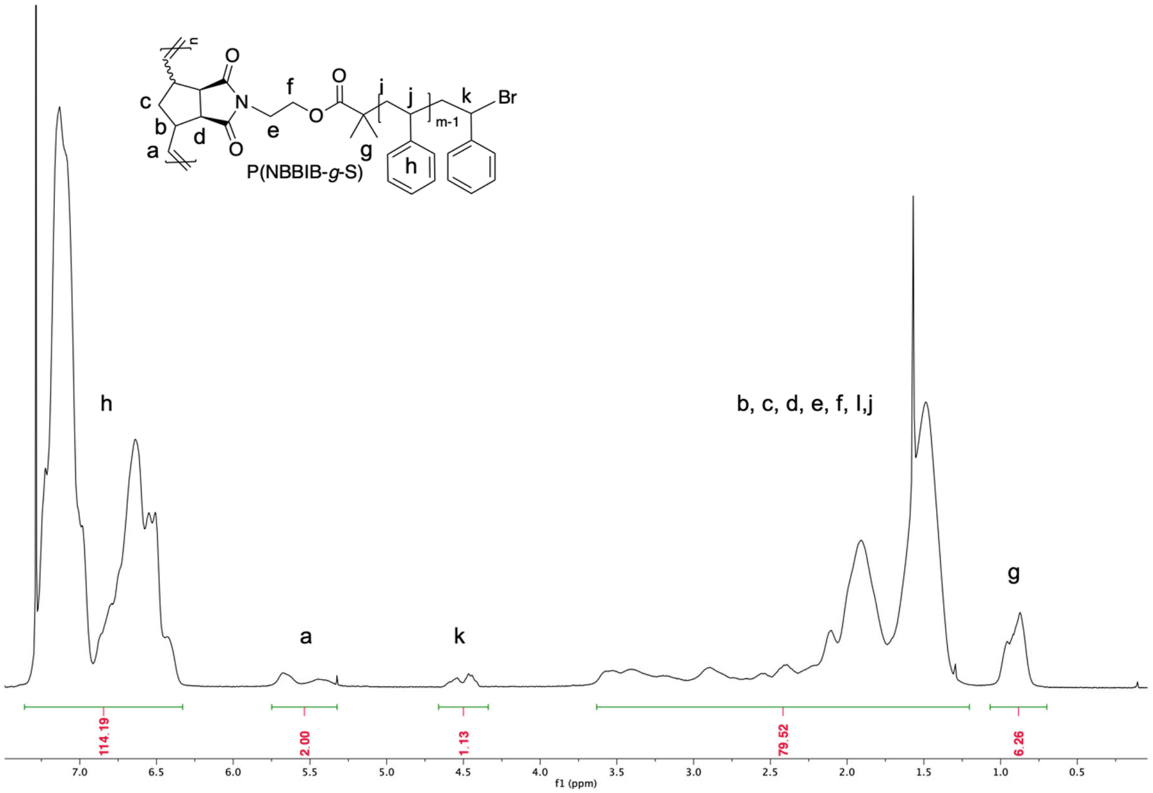

Figure S9. ^{1}H NMR (400 MHz, $CDCl_3$, 25 °C) of PNBBIB(56)-*g*-S(21) synthesized by grafting-through.

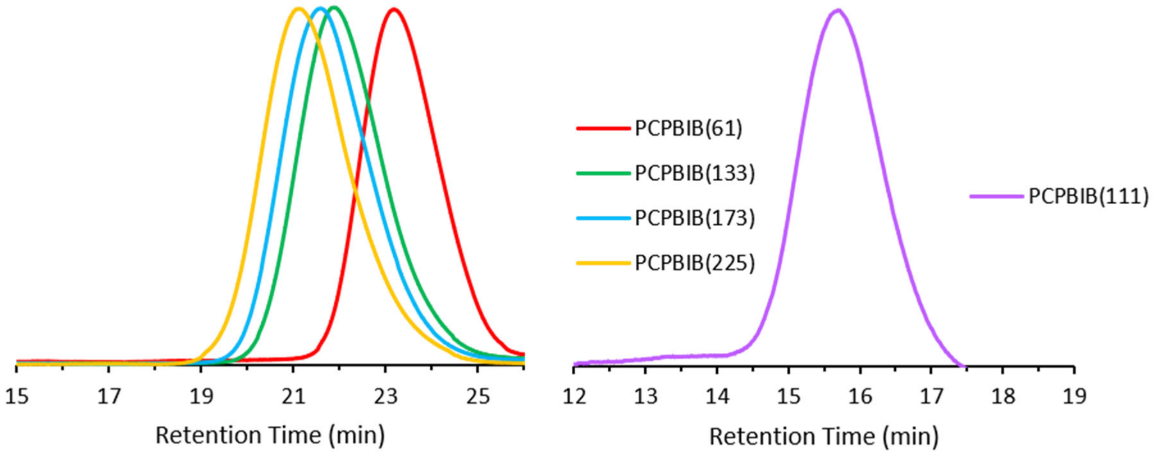

Figure S10. SEC RI traces of linear PCPBIB macroinitiators. PCPBIB(111) plotted separately due to analysis on a different SEC setup.

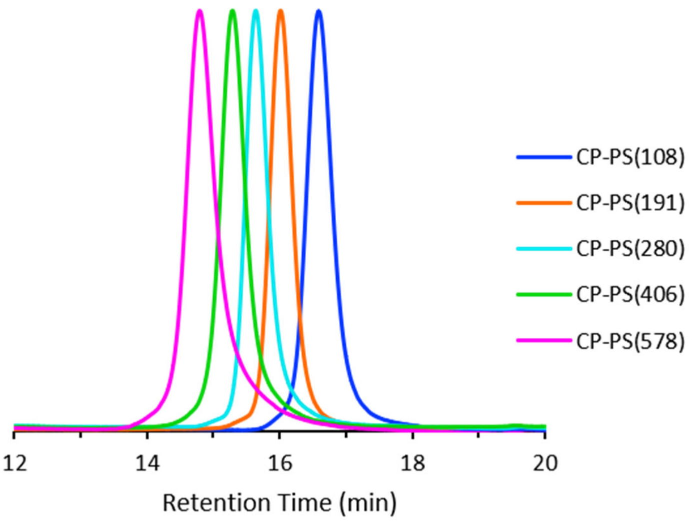

Figure S11. SEC RI traces of linear CPBIB-PS grafts.

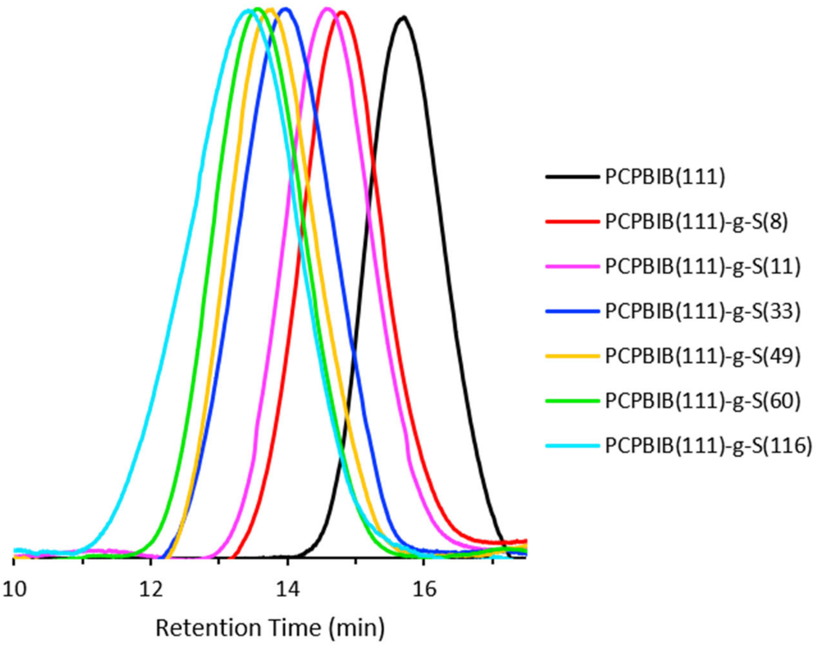

Figure S12. SEC RI traces of PCPBIB(111)-*g*-S bottlebrush series.

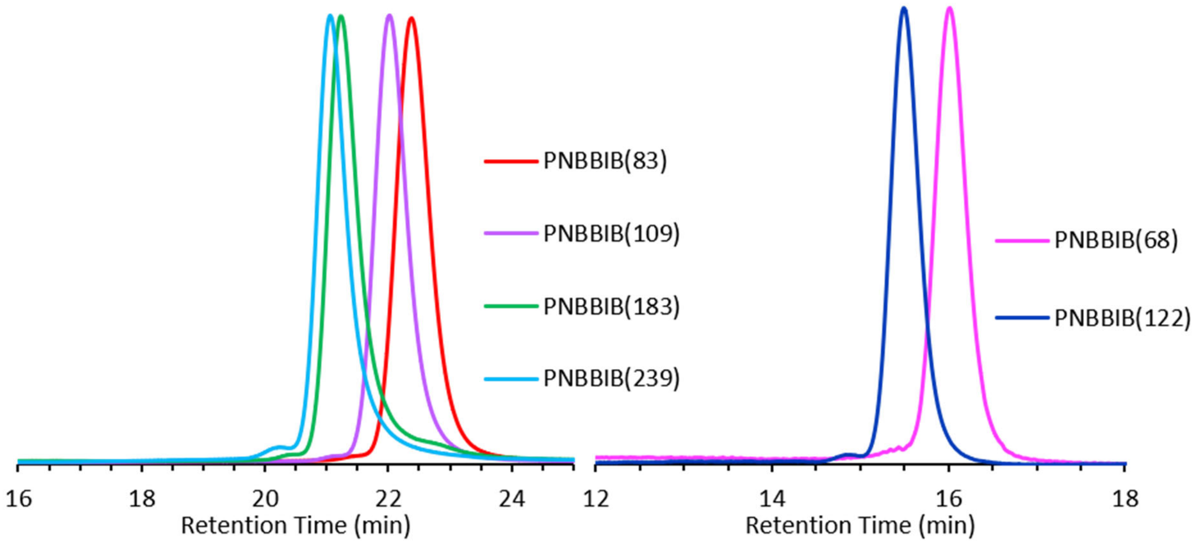

Figure S13. SEC RI traces of linear PNBBIB macroinitiators. PNBBIB(68) and PNBBIB(122) are plotted separately due to being analyzed on a different SEC setup.

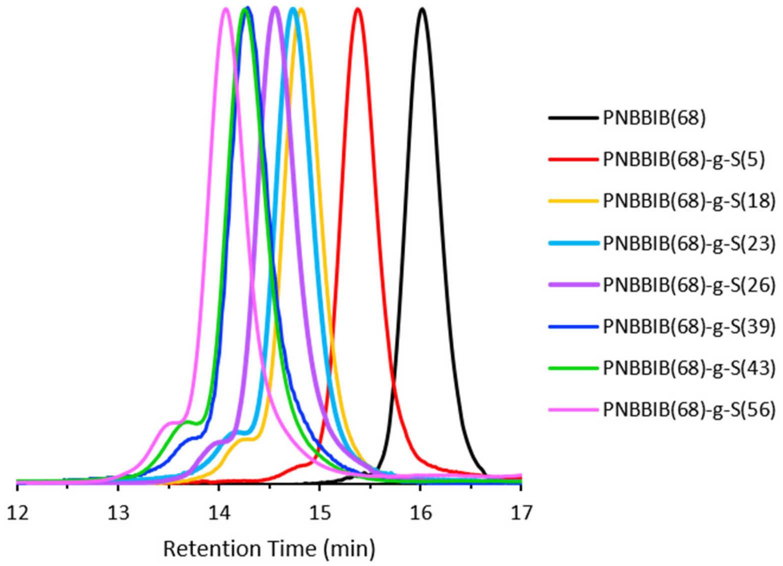

Figure S14. SEC RI traces of PNBBIB(68)-*g*-S bottlebrush series.

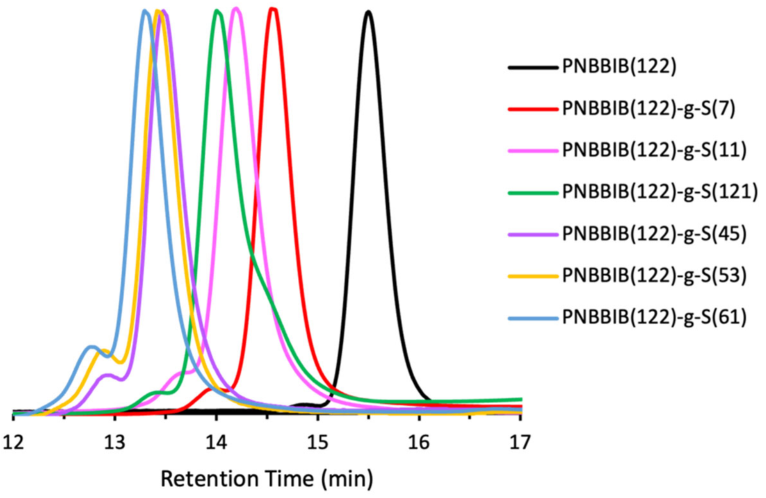

Figure S15. SEC RI traces of PNBBIB(122)-*g*-S bottlebrush series.

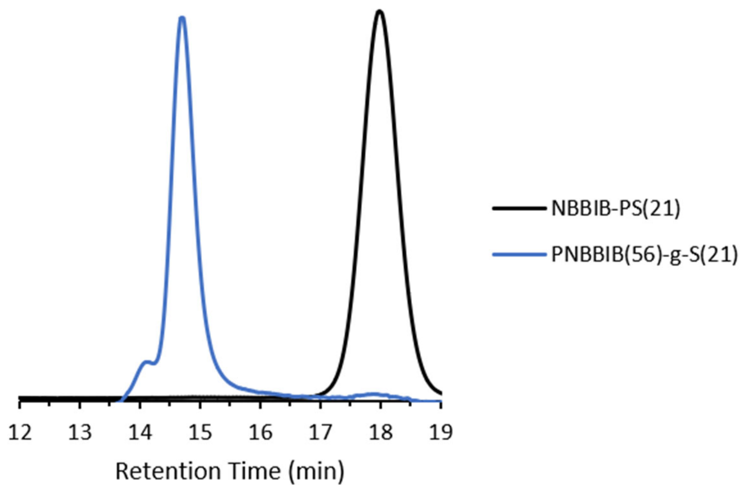

Figure S16. SEC RI traces of NBBIB-PS(21) macromonomer and PNBBIB(56)-*g*-S(21) bottlebrush synthesized from the grafting-through method.

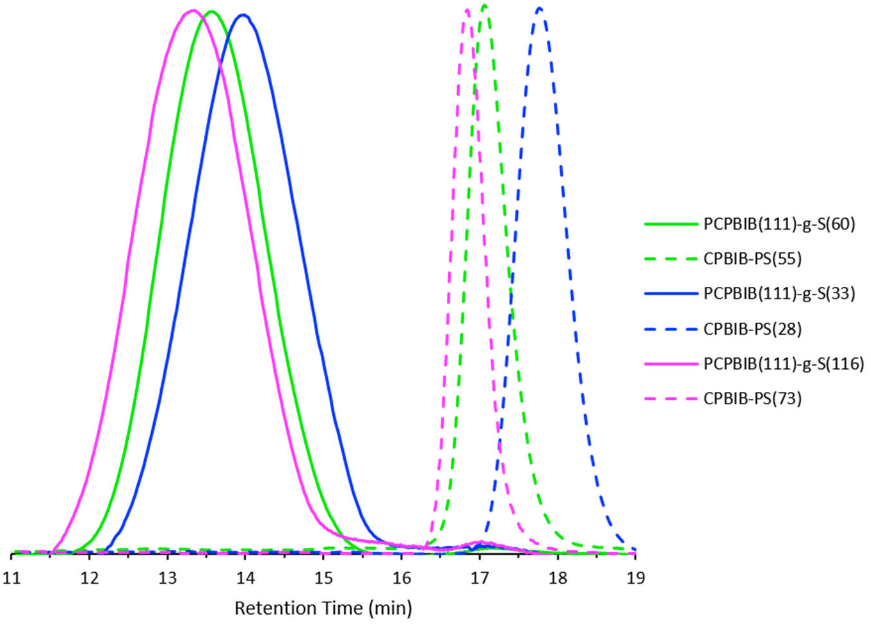

Figure S17. SEC RI traces of select PCPBIB(111)-g-S bottlebrushes (solid lines) and the respective linear CPBIB-PS grafts (dashed lines) produced after quantitative RCMD of the PCP backbone. The color of the solid line matches the color of the dashed line for the grafts produced.

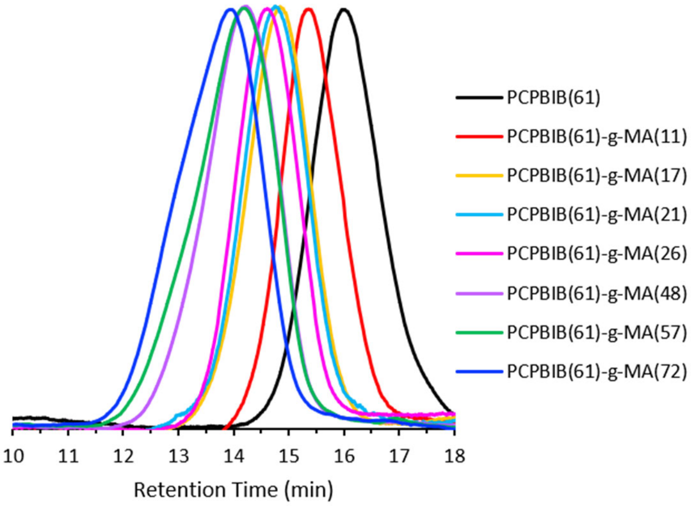

Figure S18. SEC RI traces of PCPBIB(61)-*g*-MA bottlebrush series.

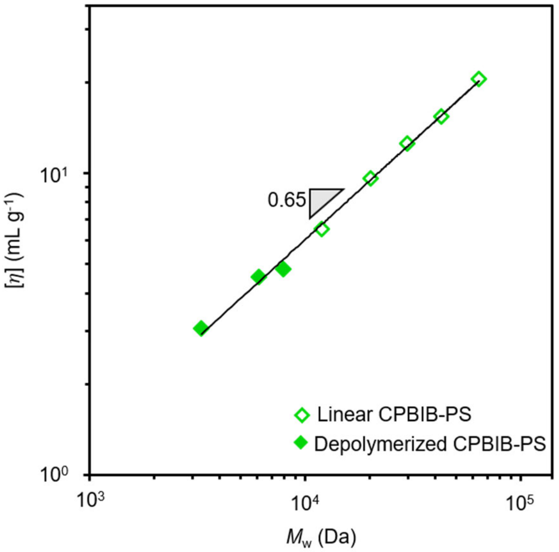

Figure S19. Intrinsic viscosity versus absolute molar mass for CPBIB-PS (open diamonds) grown directly by ATRP using CPBIB initiator and CPBIB-PS (solid diamonds) produced from RCMD of select bottlebrushes after ATRP grafting-from.

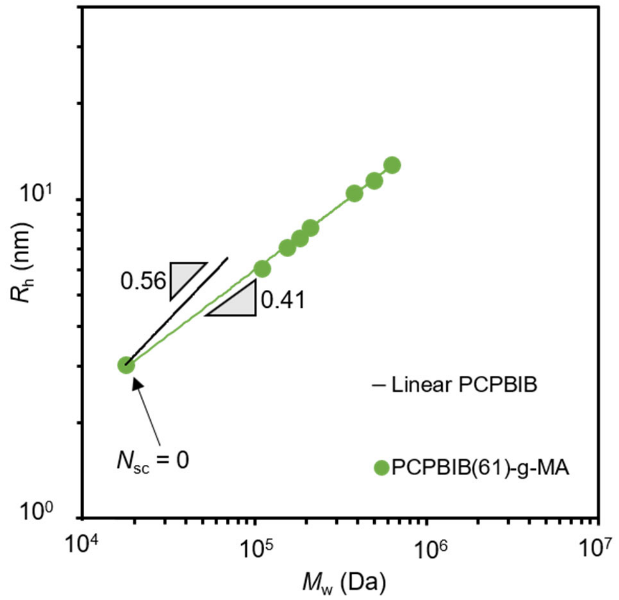

Figure S20. Hydrodynamic radius versus absolute molar mass for PCPBIB(61)-g-MA bottlebrushes. Black line represents linear PCP backbone scaling while green line is a fit to the BB data. Grey triangles are slopes and guides to the eyes.

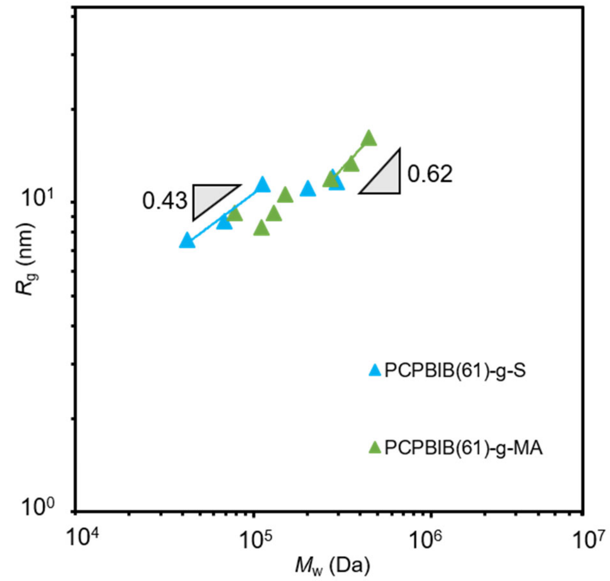

Figure S21. Radius of gyration versus absolute molar mass for PCPBIB(61)-g-MA bottlebrush series (green triangles) and comparative overlay of the PCPBIB(61)-g-S series (blue triangles). Solid lines are representative linear fits to areas of the data. Grey triangles are slopes and guides to the eyes.

Table S1. Comparative characterization data for BB sample synthesized via grafting-through and other BB samples produced from grafting-from.

Sample ID	M_n (kDa)	M_w (kDa)	Đ	R_g (nm)	R_h (nm)	[η] (mL g^{-1})
PNBBIB(56)-g-S(21)*	137.4	151.3	1.10	9.8	6.9	14.4
PNBBIB(68)-g-S(18)†	152.63	155.01	1.02	6.46	6.61	11.8
PNBBIB(68)-g-S(23)†	186.4	190.33	1.02	7.62	7.16	12.3

*Produced by grafting-through. †Produced by grafting from.

REFERENCES:

1) Neary, W. J.; Fultz, B. A.; Kennemur, J. G. *ACS Macro Lett.* **2018**, *7*, 1080.

2) Radzinski, S. C.; Foster, J. C.; Matson, J. B. *Polym. Chem.* **2015**, 6, 5643.

3) Radzinski, S. C.; Foster, J. C.; Chapleski, R. C.; Troya, D.; Matson, J. B. *J. Am. Chem. Soc.* **2016**, 138, 6998.

4) Matson, J. B.; Grubbs, R. H. *J. Am. Chem. Soc.* **2008**, 130, 6731.

5) Yarolimek M. R.; Bookbinder, H. R.; Coia, B. M. Kennemur J. G. *ACS Macro Lett.*, **2021**, *10*, 760.
